# Balanced Fluid versus 0.9% Saline in Children Treated for Septic Shock

**DOI:** 10.1056/NEJMoa2601969

**Published:** 2026-04-24

**Authors:** Fran Balamuth, Scott L. Weiss, Elliot Long, Graham C. Thompson, Amanda S. Artis, Atzael B. Campos, Meredith L. Borland, Stuart R. Dalziel, Adriana Yock-Corrales, Ruchi Singh, Amanda Williams, Beata Mickiewicz, Christopher P. Hickey, Julie C. Fitzgerald, Benjamin L. Laskin, Robert W. Hickey, Michelle Eckerle, Waleed Alqurashi, Elizabeth Alpern, Lilliam Ambroggio, Mohamed Badawy, Shannon Baumer-Mouradian, Simon Berthelot, Lindsay D. Clukies, Simon Craig, Sarah J. Curtis, Adrienne L. Davis, Susan Duffy, Matthew A. Eisenberg, Jason G. Emsley, Ara Festekjian, Shane George, Rebecca Green, Karen E. Gripp, Priya G. Jain, Shefali Jani, Gary I. Joubert, Pavan Judge, April Kam, Amit Kochar, Ioannis Koutroulis, Maria Y. Kwok, Roni D. Lane, Anna Lithgow, Julia Lloyd, Karim Mansour, Julie K. McManemy, Claudia Morris, Natalie Phillips, Arjun Rao, Alexander Rogers, Anupam Sehgal, Yasaman Shayan, Jonathan Silverman, Eunicia Tan, Neil G. Uspal, Cheryl Vance, Emma Whyte, Jing Huang, Stephen B. Freedman, Franz E. Babl, Nathan Kuppermann

**Affiliations:** 1 Department of Pediatrics, Children’s Hospital of Philadelphia, Perelman School of Medicine at the University of Pennsylvania, Philadelphia PA, USA; 2 Children’s Hospital of Philadelphia Pediatric Sepsis Program, Philadelphia PA, USA; 3 Division of Critical Care, Department of Pediatrics, Nemours Children’s Hospital, Wilmington DE, USA; 4 Departments of Pediatrics and Pathology & Genomic Medicine, Sidney Kimmel Medical Center, Thomas Jefferson University, Philadelphia PA, USA; 5 Emergency Department, The Royal Children’s Hospital, Parkville, Australia; 6 Departments of Paediatrics and Critical Care, The University of Melbourne, Parkville, Australia; 7 Murdoch Children’s Research Institute, Parkville, Australia; 8 Departments of Pediatrics and Emergency Medicine, Alberta Children’s Hospital Research Institute, Cumming School of Medicine, University of Calgary, Calgary AB, Canada; 9 Department of Biomedical and Health Informatics, Data Science and Biostatistics Unit, The Children’s Hospital of Philadelphia, Philadelphia, PA, USA; 10 Department of Biomedical and Health Informatics, Clinical Reporting Unit, Children’s Hospital of Philadelphia, Philadelphia PA, USA; 11 Department of Emergency Medicine, Perth Children’s Hospital, Nedlands, WA, Australia and School of Medicine, University of Western Australia, Australia; 12 Departments of Surgery and Pediatrics: Child and Youth Health, University of Auckland, Auckland, New Zealand and Children’s Emergency Department, Starship Children’s Hospital, Auckland, New Zealand; 13 Department of Emergency Medicine, Hospital Nacional de Niños “Dr. Carlos Sáenz Herrera”, CCSS, San José, Costa Rica; 14 Department of Anesthesiology and Critical Care, Children’s Hospital of Philadelphia, Perelman School of Medicine at the University of Pennsylvania, Philadelphia PA, USA; 15 Division of Pediatric Emergency Medicine, Department of Pediatrics, UPMC Children’s Hospital of Pittsburgh, Pittsburgh, PA, USA; 16 Cincinnati Children’s Hospital Medical Center, Cincinnati, OH; 17 Department of Pediatrics, Children’s Hospital of Eastern Ontario, University of Ottawa, Ottawa, ON; 18 Division of Emergency Medicine, Department of Pediatrics, Ann & Robert H Lurie Children’s Hospital of Chicago and Northwestern University Feinberg School of Medicine, Chicago IL USA; 19 Section of Emergency Medicine, Department of Pediatrics, Children’s Hospital Colorado and Department of Pediatrics, School of Medicine at the University of Colorado, Aurora, CO, USA; 20 Children’s Medical Center of Dallas, Dallas, TX; 21 Children’s Wisconsin, Milwaukee, WI; 22 Centre Hospitalier de l’Université Laval, Québec City, QC; 23 Division of Emergency Medicine, Department of Pediatrics, St. Louis Children’s Hospital, WashU Medicine, St. Louis MO, USA; 24 Paediatric Emergency Department, Monash Medical Centre, Melbourne, VIC, Australia and Department of Paediatrics, Sub-Faculty of Clinical and Molecular Medicine, Monash University, Melbourne, VIC, Australia; 25 Division of Pediatric Emergency Medicine, Department of Pediatrics, University of Alberta, Stollery Children’s Hospital, Women and Children’s Health Research Institute, Edmonton, Alberta, Canada; 26 Division of Emergency Medicine, Department of Paediatrics, Hospital for Sick Children and Department of Paediatrics, University of Toronto, Toronto, ON, Canada; 27 Hasbro Children’s, Providence, RI; 28 Division of Emergency Medicine, Department of Pediatrics, Boston Children’s Hospital and Departments of Pediatrics and Emergency Medicine, Harvard Medical School, Boston MA; 29 Department of Emergency Medicine, IWK Health Centre and Dalhousie University, Halifax NS, Canada; 30 Division of Emergency & Transport Medicine, Department of Pediatrics, Children’s Hospital Los Angeles, Keck School of Medicine, University of Southern California, Los Angeles, CA, USA; 31 Department of Emergency Medicine and Children’s Critical Care, Gold Coast University Hospital, Southport, QLD, Australia and School of Medicine and Dentistry, Griffith University, Southport, QLD, Australia; 32 Section of Pediatric Emergency Medicine, Department of Pediatrics and Child Health, HSC Winnipeg Children’s Hospital and Max Rady College of Medicine, Rady Faculty of Health Sciences, University of Manitoba, Winnipeg MB, Canada; 33 Department of Emergency Medicine, The Children’s Hospital at Westmead, Westmead NSW, Australia; 34 London Health Sciences Centre, London, ON; 35 British Columbia Children’s Hospital, Vancouver, BC; 36 McMaster Children’s Hospital, Hamilton, ON; 37 Department of Emergency Medicine, Women’s and Children’s Hospital, Adelaide SA, Australia and Department of Acute Care Medicine, University of Adelaide, Adelaide, SA, Australia; 38 Division of Emergency Medicine, Department of Pediatrics, Children’s National Hospital and George Washington University School of Medicine and Health Sciences, Washington, DC, USA; 39 Department of Emergency Medicine, Columbia University, Vagelos College of Physicians and Surgeons, New York-Presbyterian Morgan Stanley Children’s Hospital, New York, NY, USA.; 40 Division of Emergency Medicine, Primary Children’s Hospital, Department of Pediatrics, University of Utah School of Medicine, Salt Lake City, Utah, USA; 41 Department of Paediatrics, The Royal Darwin Hospital Tiwi, NT, Australia; 42 Nationwide Children’s Hospital, Columbus, OH; 43 University of California San Francisco, Benioff Children’s Hospital, Oakland, CA; 44 Division of Emergency Medicine, Department of Pediatrics, Texas Children’s Hospital and Department of Pediatrics, Baylor College of Medicine, Houston, TX, USA; 45 Emory Children’s Healthcare of Atlanta, Atlanta, GA; 46 Emergency department, Queensland Children’s Hospital, South Brisbane, QLD, Australia and Child Health Research Centre, University of Queensland, Brisbane, QLD, Australia; 47 Department of Emergency Medicine, Sydney Children’s Hospital, Randwick, NSW, Australia and School of Women’s and Children’s Health, University of New South Wales, Kensington, NSW, Australia; 48 Departments of Emergency Medicine and Pediatrics, University of Michigan, Ann Arbor, Michigan; 49 Kingston Health Sciences Centre, Kingston, ON; 50 Centre Hospitalier Universitaire Sainte Justine, Montréal, QC; 51 Children’s Hospital of Richmond at Virginia Commonwealth University, Richmond, VA; 52 Middlemore Hospital, Auckland, New Zealand and Department of Surgery and Paediatrics: Child and Youth Health, The University of Auckland, Auckland, New Zealand; 53 Seattle Children’s Hospital, Seattle, WA; 54 University of California Davis Children’s Hospital, Sacramento, CA; 55 Emergency Department, Townsville University Hospital, Douglas, QLD, Australia and College of Medicine and Dentistry, James Cook University, Townsville, QLD, Australia; 56 Department of Biostatistics, Epidemiology, and Informatics, Perelman School of Medicine at the University of Pennsylvania, Philadelphia, PA, USA; 57 Sections of Pediatric Emergency Medicine and Gastroenterology, Departments of Pediatrics and Emergency Medicine, Cumming School of Medicine, University of Calgary, Calgary AB, Canada; 58 Departments of Pediatrics and Emergency Medicine, George Washington University School of Medicine and Health Sciences, and Children’s National Hospital, Washington, DC, USA

## Abstract

**Background:**

Whether treatment with balanced crystalloid fluid improves outcomes compared with 0.9% saline for children treated for septic shock is debated.

**Methods:**

This pragmatic clinical trial, conducted in 47 emergency departments across five countries, randomly assigned patients 2 months to <18 years with suspected septic shock and abnormal perfusion to either balanced fluid or 0.9% saline for resuscitation and maintenance fluids for up to 48 hours. Primary outcome was a major adverse kidney event (MAKE30), a composite of death, new renal replacement therapy, or persistent kidney dysfunction, at 30 days following enrollment or hospital discharge, whichever occurred first.

**Results:**

Of 9,041 patients enrolled, 559 (6.2%) withdrew without data use, leaving 4,235 assigned to balanced fluid and 4,247 assigned to 0.9% saline for analysis. The primary outcome, MAKE30, occurred in 137 (3.4%) in the balanced fluid and 124 (3.0%) in the 0.9% saline group (absolute difference, 0.4 percentage points; 95% CI, −0.5 to 1.3; risk ratio, 1.10; 95% CI, 0.88 to 1.40, P=0.85). Components of the primary outcome were also not different between groups. Both groups had 23 median hospital-free days of 28 (interquartile range, 19–25). Hyperchloremia occurred in 866 (31%) and 1,378 (49%) of the balanced fluid and saline groups, respectively; hypernatremia in 52 (1.9%) and 89 of 2,862 (3.1%), respectively; hyperlactatemia in 259 (20%) and 228 (17%), respectively. Other adverse events were similar between groups. No differences in other safety outcomes or adverse events were seen.

**Conclusions:**

Among children treated for septic shock with fluid resuscitation, balanced fluid compared with 0.9% saline showed no significant difference in the composite outcome of death, new renal replacement therapy, or persistent kidney dysfunction. (The study was registered at ClinicalTrials.gov (NCT04102371) on September 25, 2019.)

Crystalloid fluid is commonly used to resuscitate children with septic shock.^1^ Options for crystalloids include 0.9% sodium chloride (saline) or balanced fluid, such as lactated Ringer’s (LR) or Plasma-Lyte^™^. Despite its widespread use,^2,3^ 0.9% saline contains a supra-physiologic concentration of chloride that is associated with hyperchloremia, metabolic acidosis, and decreased renal blood flow.^4,5^ In contrast, balanced fluid contains electrolyte compositions more closely resembling human plasma^6^ and has been associated with lower frequencies of acute kidney injury (AKI), renal replacement therapies (RRT), and death compared with 0.9% saline in some adult^7–14^ and pediatric^15–17^ studies. However, others have reported no benefit^18–22^ or harm.^23^ Consequently, in 2020 the Surviving Sepsis Campaign issued a conditional recommendation for balanced fluids over 0.9% saline in children with septic shock.^24^

To determine if resuscitation with balanced fluid for children with septic shock would result in a lower frequency of major adverse kidney events within 30 days (MAKE30) compared with 0.9% saline, we conducted the Pragmatic Pediatric Trial of Balanced versus Normal Saline Fluid in Sepsis (PRoMPT BOLUS). We hypothesized that resuscitation and maintenance hydration with balanced fluid would decrease the rate of MAKE30 compared with 0.9% saline.

## METHODS

### Trial Design and Oversight

We conducted a multicenter, pragmatic, open-label, randomized interventional trial in which children with suspected septic shock treated in an emergency department (ED) between August 25, 2020, and October 31, 2025, were allocated to balanced crystalloids or 0.9% saline for resuscitation and maintenance fluids. The study was conducted at 47 sites in the United States (US), Canada, Australia, New Zealand, and Costa Rica. Human subjects regulatory oversight was administered separately within each country (see Supplementary Appendix). The protocol demonstrated feasibility in a pilot study,^25^ and methodologic details were previously published.^26^ The protocol and statistical analysis plan are available at NEJM.org.

The first and last authors designed the study and wrote the initial draft of the manuscript. The first authors and biostatisticians (AA, JH) had full access to the data, vouch for it and analyzed and confirmed the data independently. An international steering committee oversaw all study activities, including data collection, and vouches for data accuracy and fidelity of protocol adherence. All authors reviewed the final version of the manuscript and participated in the decision to publish. The sponsors had no role in the design or conduct of the study, data analysis, or approval of the manuscript.

### Study Participants

Patients from 2 months to <18 years-old being treated in an ED for suspected septic shock with fluid resuscitation for abnormal perfusion were eligible for enrollment provided that total volume of crystalloid fluid administration was confirmed as ≤40 mL/kg prior to enrollment. All participating ED clinicians screened patients during routine care, but only those trained on study procedures could enroll. Patients could be enrolled at any time prior to ED disposition so long as eligibility criteria were met. Enrolled patients could participate more than once. Patients for whom the treating clinician judged it unsafe to administer either fluid type were excluded (Supplementary Appendix for additional details).

Due to the narrow therapeutic window to begin fluid resuscitation, enrollment adhered to “Exception from Informed Consent” (21 CFR 50.24) for emergency research in the US^27^ and ethically-approved alternative processes to prospective informed consent in Canada, Australia/New Zealand, and Costa Rica, as described in the Supplementary Appendix.^28,29^

### Randomization

Study patients were allocated to either balanced fluid or 0.9% saline using permuted-block randomization, stratified by site (Supplementary Appendix). Treatment allocation was concealed using serially numbered, opaque envelopes for efficiency of enrollment concurrent with clinical management. Fluid type allocation was revealed after eligibility was confirmed and the patient was enrolled.

### Intervention

We compared treatment with a predominantly balanced fluid or 0.9% saline strategy, acknowledging that routine care often includes multiple fluid types (Supplementary Appendix, Fig. S1). Balanced fluids could be LR, Plasma-Lyte^™^, or Hartmann’s solution, depending on availability or clinician preference (Table S1), but the allocated study fluid was preferentially used for all bolus fluid and as the base fluid for maintenance hydration until 11:59 P.M. of the following day (Table S2). The intervention phase was timed to end so that all patients would receive study fluid for 24 to 48 hours, the time-window when most fluid resuscitation for septic shock is completed and which provided a pragmatic end to the intervention phase.^14^

Each site established procedures to promote protocol adherence (Supplementary Appendix). Other than fluid type, all decisions about timing, volume, and rate of fluid administration remained at the discretion of the treating clinicians. Alternative fluid types were allowed for clinical indications (e.g., hyponatremia). Maintenance fluids were included because these constitute a substantial proportion of total crystalloid fluid administration.^25,30^ Non-isotonic fluids (e.g., 0.45% saline) are not recommended as maintenance fluid in children and were discouraged.^31,32^ Hospital fluid supplies were used without changes to labeling.

Patients and clinicians were not blinded to treatment allocation as a practical necessity and to reduce risk that clinicians might attribute electrolyte changes to the study fluid and stop adhering to the protocol. However, the senior biostatistician (JH) and all investigators remained blinded to aggregate outcomes until enrollment was complete.

### Data Collection

Data obtained during clinical care were extracted from the medical record (Supplementary Appendix) and monitored for quality within each network. A central Data Coordinating Center at CHOP collated data across networks for interim and final analyses. Site of infection and diagnosis of septic shock were ascertained by the lead investigator at each site using all available data. AKI at presentation was determined using serum creatinine cut-points recommended by Kidney Disease Improving Global Outcomes (KDIGO) guidelines.^33,34^

### Outcomes

The primary outcome was one or more criteria for major adverse kidney events at 30 days (MAKE30)—a composite of death from any cause, initiation of RRT, or persistent kidney dysfunction—following study enrollment or hospital discharge, whichever occurred first.^35^ RRT included treatment with any renal replacement modality started during the hospitalization. Persistent kidney dysfunction was defined as a final serum creatinine at least 200% of baseline *and* a minimum increase of at least 0.3 mg/dL. Baseline serum creatinine was recorded as the lowest value available between 12 months and 24 hours prior to enrollment or, if missing, imputed using median creatinine values for age and sex (Supplementary Appendix).^36^

Secondary effectiveness outcomes included components of MAKE30, hospital length of stay, hospital-free days out of 28, and all-cause mortality prior to hospital discharge and within 90 days after randomization. Safety outcomes included electrolyte abnormalities within three days of enrollment, arterial/venous thrombosis, and cerebral edema diagnosed during clinical care. Adverse events were collected for seven days after enrollment (Supplementary Appendix).

### Statistical Analysis

Details regarding sample size determination were previously published.^26^ Briefly, enrollment of 8,800 participants was calculated as providing 95% power to detect an absolute risk reduction in MAKE30 from 6.0% for children treated with 0.9% saline (based on preliminary data^36^) to 4.3% for balanced fluid with type-I error of 0.05. To account for withdrawal after EFIC/deferred consent, the final enrollment target was increased by 4% to 9,178.

The primary analysis included all randomized patients, except those who withdrew use of their data. Patients later determined not to have met eligibility criteria were retained in the primary analysis to avoid bias from differential assessment of eligibility. Multiple imputation was used to account for missing outcome data, with the assumption that data were missing-at-random (Supplementary Appendix).

MAKE30 and other binary outcomes were compared using the Cochran–Mantel–Haenszel test, stratified by study site. Continuous outcomes were compared using the Van Elteren test, stratified by site. Mortality within 90 days was estimated with the Kaplan–Meier method, and a mixed-effects Cox proportional-hazards model was used to compare groups, with random intercepts for study site. The proportional-hazards assumption was assessed by visual inspection of log–log survival plots and by testing Schoenfeld residuals, and was not violated.

Sensitivity analyses included patients with complete primary outcome data and a per-protocol analysis of patients who received at least 75% of their total crystalloid fluid volume during the intervention phase as the fluid to which they were randomized. We also conducted a tipping-point analysis to assess the robustness of the primary analysis of MAKE30 to departures from the missing-at-random assumption. For safety outcomes with more than 30% missing data, additional sensitivity analyses were performed (Supplementary Appendix).

Prespecified subgroup analyses were conducted for age, sex, cancer comorbidity, AKI at presentation, total fluid volume during the intervention phase, and country of enrollment (Supplementary Appendix). Additional post-hoc analyses evaluated measured versus imputed baseline creatinine and initial severity of acidosis and hyperlactatemia. Subgroup analyses were performed using multiply imputed data.

A data and safety monitoring board (DSMB) oversaw the study. Interim analyses for efficacy were performed after enrollment of 15%, 40%, and 70% of participants using a group sequential design with symmetric two-sided O’Brien-Fleming boundaries to control the overall type I error rate at 0.05. A two-sided P-value of less than 0.044 at the final analysis indicated statistical significance for the primary outcome. Due to multiplicity, statistical significance is not reported for secondary effectiveness outcomes, and unadjusted P-values are reported for safety outcomes. All analyses were performed using SAS, Version 9.4 (SAS Institute, Cary, NC) or R, Version 4.4.1 (R Foundation for Statistical Computing, Vienna, Austria).

## RESULTS

### Study Participants

Of the 9,041 participants enrolled, 4,512 were randomized to balanced fluid and 4,529 to 0.9% saline (Figs. S2–S4 and Table S3). A total of 277 (6.1%) and 282 (6.2%) from the balanced fluid and 0.9% saline groups withdrew use of their data. Therefore, the analysis included 4,235 patients assigned to balanced fluid and 4,247 assigned to 0.9% saline.

### Baseline Characteristics

Patient characteristics were similar between groups (Tables 1 and S4–S5). Median age was 6.8 years (interquartile [IQR], 2.8–13), and 50.1% were male. The site of infection was most often respiratory (46.6%, Table S6), and only 5.8% were determined not to have sepsis (Table S7). At presentation, 1,172 (13.8%), 415 (4.9%), and 432 (5.1%) had stage 1, 2, or 3 AKI, respectively (Tables 1 and S8).

Treatments for sepsis through the intervention phase are summarized in Table S9. Overall, 1,207 (14.2%) received vasoactive medications and 822 (9.7%) required invasive mechanical ventilation. Treatment with bicarbonate occurred in 5.2%, including 194 (4.6%) in the balanced fluid and 249 (5.9%) in the 0.9% saline groups.

### Fluid Administration

**Figure 1:**
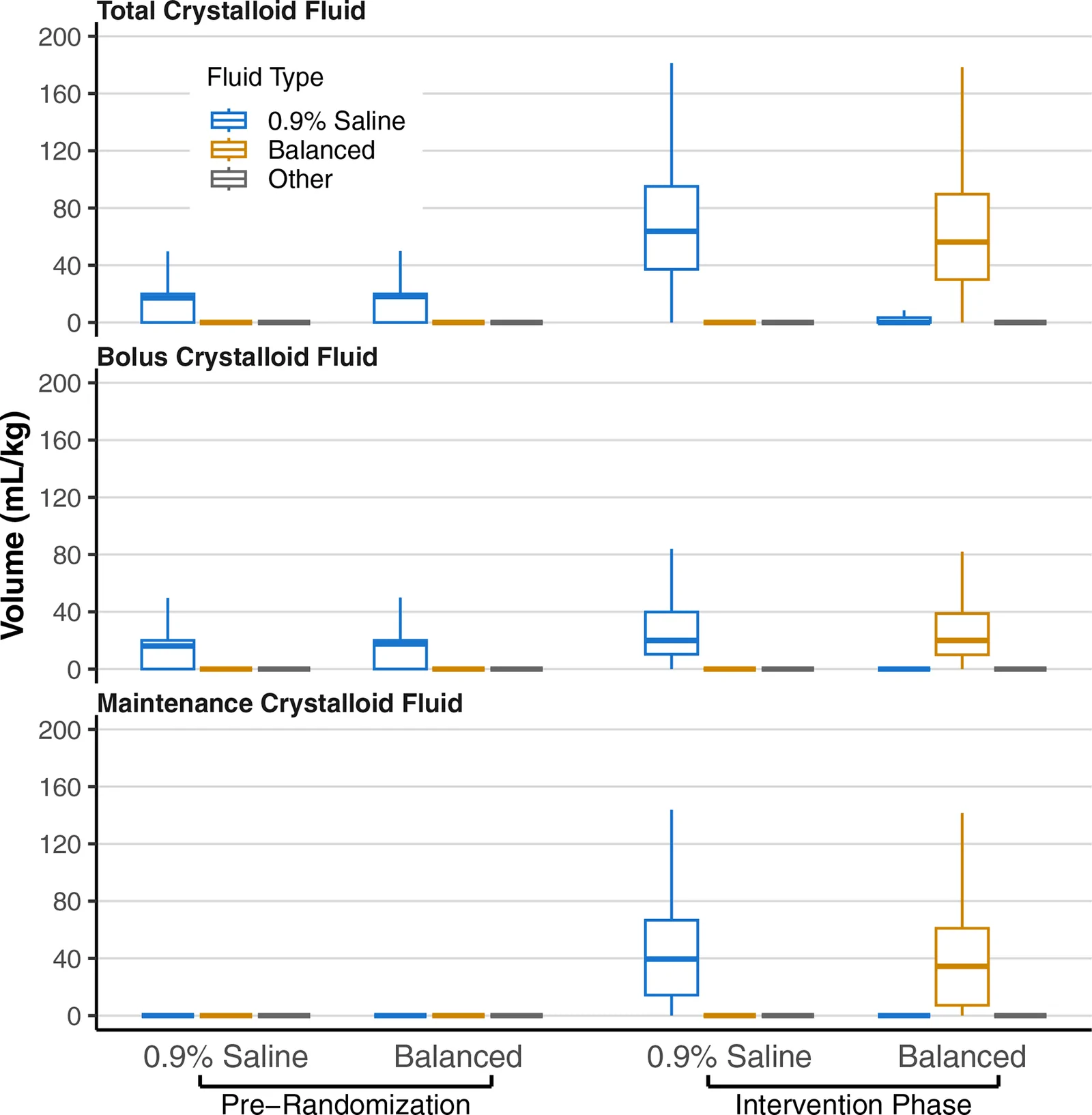
Boxplot of Type and Volume of Fluid Administration Shown are the volumes of crystalloid fluid type received by patients according to assigned treatment group of balanced fluid or 0.9% saline. The data shown are the total (Panel A), bolus (Panel B), and maintenance (Panel C) crystalloid volumes received starting from ED presentation until 11:59 pm on the calendar day after the day of randomization. Maintenance fluids were categorized as the base fluid used regardless of additives (e.g., dextrose, potassium). Data are presented as boxplots with median, interquartile range, and whiskers indicating values +/− 1.5 times the 25% and 75% percentiles, respectively (outliers beyond these thresholds are not shown).

The volumes of total, bolus, and maintenance crystalloid fluids were similar between treatment groups prior to randomization and during the interventional phase (Fig. 1). The median total crystalloid volume, including both prior to randomization and during the intervention phase, was 85 mL/kg (IQR, 55–119) and 88 mL/kg (IQR, 57–123) for the balanced fluid and 0.9% saline groups, respectively (difference in medians, −2.3 mL/kg; 95% CI −4.3 to −0.3;Table S10 and Fig. S5).

The median volume of 0.9% saline received was 20 mL/kg (IQR, 5.5–32) in the balanced fluid group compared with 79 mL/kg (IQR, 49–113) in the 0.9% saline group, while the median volume of balanced fluid was 58 mL/kg (IQR, 31–92) in the balanced group compared with 0 mL/kg (IQR, 0–0) in the 0.9% saline group (Table S10). Overall, 80% of patients in the balanced fluid and 88% in the 0.9% saline groups received at least 75% of total crystalloid as their randomized fluid type.

### Primary Outcome

MAKE30 occurred in 137 of 4,073 patients (3.4%) in the balanced fluid group and 124 of 4,068 (3.0%) in the 0.9% saline group (absolute difference, 0.4 percentage points; 95% CI, −0.5 to 1.3; risk ratio, 1.10; 95% CI, 0.88 to 1.40, P=0.85; Table 2 and Fig. S6). The results were similar in pre-specified sensitivity analysis of patients without missing components of MAKE30 and the per-protocol analysis (Table S11). A post-hoc analysis did not demonstrate effect modification from pre-randomization 0.9% saline administration (Table S12). Tipping-point analysis demonstrated that the findings for MAKE30 remained robust to all but extreme departures from the missing-at-random assumption (Fig. S7).

### Secondary Outcomes

Results for secondary effectiveness and safety outcomes are shown in Table 2. Median hospital-free days out of 28 were 23 (19–25) in both groups. Mortality was not different between groups at hospital discharge or within 90 days (Table 2 and Fig. S8).

Among patients with follow-up laboratory values measured through study day 3, hyperchloremia and hypernatremia occurred less often, and hyperlactatemia more often, with balanced fluid (Tables 2 and S13 and Fig. S9). The lower rate of hyperchloremia with balanced fluid remained significant across sensitivity analyses (Table S14 and Fig. S10). Differences in follow-up blood chloride, bicarbonate, and creatinine were more pronounced between groups as crystalloid volume increased (Figs. S11–13). Thrombosis and cerebral edema did not differ between groups (Table 2), nor did other adverse events (Table S15).

### Subgroup Analyses

**Figure 2:**
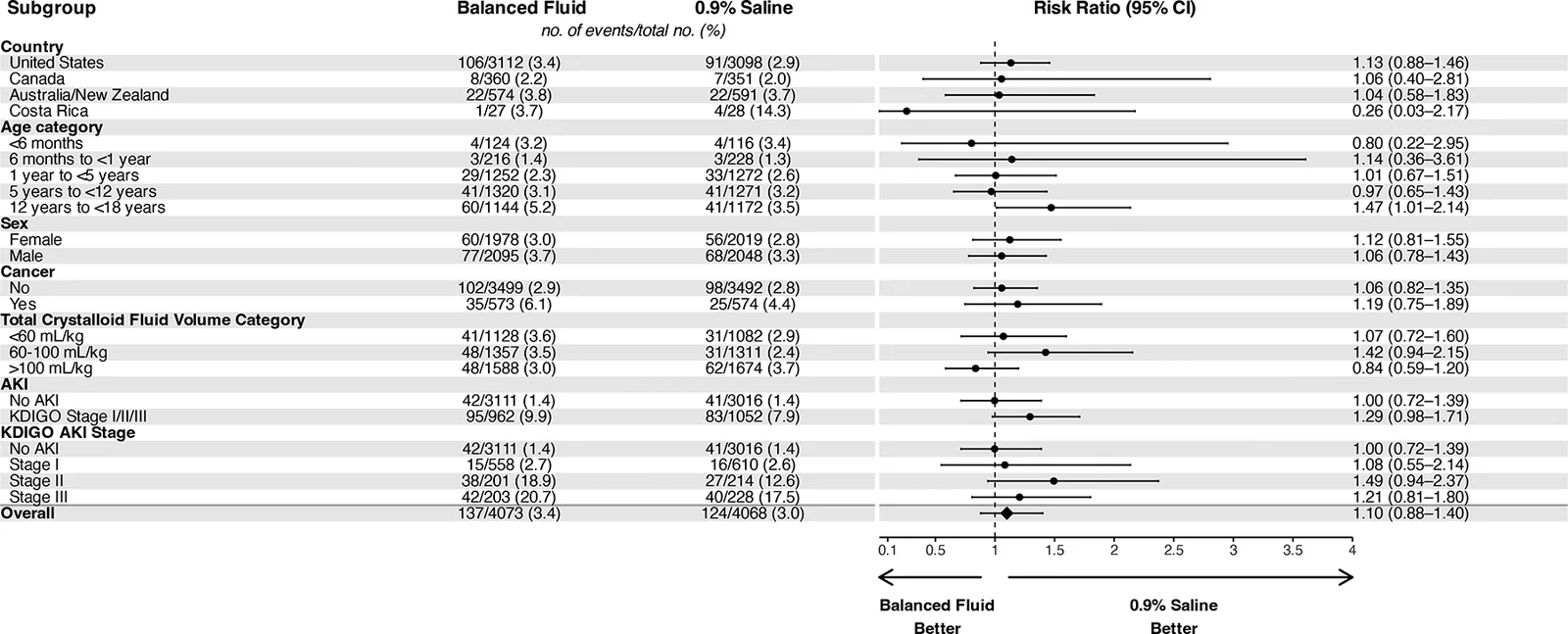
Subgroup Analyses The risk ratio and 95% confidence interval are shown according to subgroup for the percentage of patients in the balanced fluid and 0.9% saline groups who met criteria for the composite outcome of a major adverse kidney event within 30 days (defined as death from any cause, new renal replacement therapy, or persistent kidney dysfunction). Cancer was present if the patient was being treated for or monitored with a blood or solid tissue cancer prior to study enrollment or starting during the study-related hospitalization but did not include remote cancer diagnoses considered cured or resolved. Total crystalloid fluid volume refers to the total volume of crystalloid received during the combined pre-randomization and interventional phases as either bolus fluid or maintenance hydration. Acute kidney injury (AKI) refers to the Kidney Disease Improving Global Outcomes (KDIGO) classification (see Supplementary Appendix).

MAKE30 did not differ between balanced fluid and 0.9% saline within pre-specified subgroups (Fig. 2) or post-hoc analyses stratified by measured versus imputed baseline creatinine (Fig. S14) or initial bicarbonate or lactate levels (Fig. S15). A post-hoc analysis comparing MAKE30 across increasing total volumes of crystalloid fluid also did not demonstrate differences between treatment groups (Fig. S16).

## DISCUSSION

In this trial of children treated for suspected septic shock in an emergency department, use of balanced fluid for up to 48 hours did not reduce MAKE30, hospital-free days, or mortality. Adequate treatment separation was evident in the distribution of fluid type between groups, as well as by lower rates of hyperchloremia and hypernatremia in the balanced fluid group. Hyperlactatemia was also more frequent with balanced fluids, but there were no differences in rates of thrombosis, cerebral edema, or other adverse events.

Our data do not show benefit for the routine use of balanced fluid over 0.9% saline in children with suspected septic shock. As in prior studies, balanced fluid resulted in less hyperchloremia and hypernatremia,^6,16^ but these biochemical effects did not translate to improved patient-centered outcomes. Notably, our findings differ from the trial by Sankar et al. which found a 38% relative reduction in new/progressive AKI with balanced fluid among 708 children with septic shock.^16^ However, in that study, the outcome was only monitored for seven days and included any increase in serum creatinine of 0.3 mg/dL or more, whereas MAKE30 required at least a two-fold increase above baseline creatinine. Despite these differences, neither Sankar et al.^16^ nor our study found differences in mortality or hospital-free days.

A meta-analysis of six clinical trials in adult patients estimated an 89.5% probability that balanced fluids reduce mortality compared to 0.9% saline.^37^ However, a recent cluster-randomized trial of 43,626 patients did not find a beneficial effect of hospital-wide use of LR.^19^ Two prior adult studies demonstrated small, but significant, reductions in MAKE30, but neither demonstrated differences in hospital-free days.^13,14^

Strengths of our trial included a large sample size, which was needed to detect small differences in patient outcomes. Additionally, we only enrolled patients prior to large-volume fluid resuscitation because prior studies had suggested that this population would be most likely to benefit from balanced fluid.^14,21^

Our study also had several limitations. First, it is not certain that our results are generalizable to low-resource settings or hospital-acquired sepsis. Second, we defined septic shock using immediately accessible signs of abnormal perfusion to capture patients near the onset of fluid therapy rather than wait for higher-risk criteria, which are often not available at presentation. Although this approach aligns with clinical practice and the study population was representative of children treated for septic shock in an ED (Table S16), the low event rate diminished statistical power to detect planned differences between groups. Moreover, while we did not observe heterogeneity of treatment effect in subgroup analyses, point estimates favoured balanced fluid among patients who received the highest fluid volumes and presented with more extreme acidosis and hyperlactatemia; thus, we cannot exclude a benefit of balanced fluid in children with the most severe illness. Third, although studies have shown that most crystalloid fluid is administered within the initial 48 hours of sepsis treatment,^6,14,16,21^ it is possible that unmeasured fluid administration after the intervention phase altered the outcomes. Fourth, our analysis could not distinguish patients enrolled more than once. Fifth, a higher proportion of patients withdrew prior to ascertaining the primary outcome than expected. However, withdrawal was unlikely to be related to the intervention, and the results were robust to all but unplausible conditions among patients with missing data. Finally, MAKE30 is composed of endpoints that may not be of equivalent value to patients; however, there were no differences in the sub-components or other secondary effectiveness outcomes.

In this pragmatic, randomized clinical trial of approximately 9,000 children with suspected septic shock treated with fluid resuscitation for abnormal perfusion in an ED, there was no significant difference between balanced fluid and 0.9% saline in the composite outcome of death, new renal replacement therapy, or persistent kidney dysfunction.

## Supplementary Material

supplment

## Figures and Tables

**Table 1:** Patient Characteristics

Characteristic	Balanced Fluid	0.9% Saline
	(n =4,235)	(n = 4,247)
Age in years - median [IQR]	6.8 [2.8, 13]	6.8 [2.8, 13]
Missing	3	5
Male sex – no. (%)	2,166 (51)	2,136 (50)
Missing	3	5
Comorbid Conditions – no. (%)		
Cancer (hematogenous or solid tumor)	603 (14)	613 (14)
Bone marrow or solid organ transplant	165 (3.9)	158 (3.7)
Cardiomyopathy or heart failure	43 (1.0)	47 (1.1)
Pulmonary hypertension	28 (0.66)	30 (0.71)
Kidney disease	193 (4.6)	175 (4.1)
Neurologic dysfunction causing severe developmental delay	1,182 (28)	1,182 (28)
Sickle cell disease	61 (1.4)	59 (1.4)
Chronic ventilator dependence	247 (5.8)	219 (5.2)
Indwelling central catheter	745 (18)	742 (18)
Missing	11	11
Minutes from time of emergency department arrival to first antibiotic administration – median [IQR]^c^	94 [50, 183]	90 [48, 183]
Minutes from time of emergency department arrival to study enrollment – median [IQR]^d^	103 [50, 191]	101 [49, 188]
Crystalloid fluid received prior to enrollment (mL/kg) – median [IQR]		
0.9% saline	18 [0, 20]	17 [0, 20]
Balanced fluid	0 [0, 0]	0 [0, 0]
Initial blood lactate (mmol/L) – median [IQR]^e^	1.9 [1.3, 3.0]	1.9 [1.3, 3.0]
Not measured	1,634	1,652
Baseline creatinine (mg/dL) – median [IQR]^f^	0.30 [0.25, 0.50]	0.30 [0.25, 0.47]
Missing	3	4
KDIGO acute kidney injury stage at enrollment – no. (%)		
Stage 1	560 (13)	612 (14)
Stage 2	201 (4.7)	214 (5.0)
Stage 3	204 (4.8)	228 (5.4)

KDIGO, Kidney Disease: Improving Global Outcomes

b Patients found to be 18 years-old after enrollment were retained in the primary analysis to avoid bias from differential assessment of eligibility

c Includes the subset of participants (n = 8,157) who did not receive first antibiotic prior to study site ED arrival

d Minutes from time of emergency department arrival to study enrollment excludes 31 patients (16 in the balanced fluid group and 15 in the 0.9% saline group) with invalid time intervals from erroneous timestamps

e Initial blood lactate was the value measured closet to study enrollment between 6 hours before through 2 hours after randomization

f Baseline serum creatinine was measured as the lowest creatinine available between 12 months and 24 hours prior to study enrollment in 5,044 (59.5%) patients and imputed using established median values for age and sex in 3,431 (40.5%) patients (missing data occurred in 7 patients because age and/or sex was also missing)

**Table 2:** Outcomes

Outcome^a^	Balanced Fluid	0.9% Saline	Risk Difference (95% CI)^b^	Effect Measure (95% CI)^c^	*P* ^d^
**Primary outcome**					
Major adverse kidney events within 30 days – no./total no. available (%)	137/4,073 (3.4)	124/4,068 (3.0)	0.004 (−0.005 to 0.013)	1.10 (0.88 to 1.40)	0.85
**Components of the primary outcome**					
Death within 30 days – no./total no. available (%)	41/4,214 (0.97)	39/4,226 (0.92)	0.001 (−0.004 to 0.005)	1.10 (0.70 to 1.60)	
New renal replacement therapy – no./total no. available (%)	26/4,214 (0.62)	31/4,226 (0.73)	−0.001 (−0.005 to 0.002)	0.84 (0.50 to 1.40)	
Persistent kidney dysfunction at hospital discharge – no./total no. available (%)	93/4,085 (2.3)	78/4,079 (1.9)	0.004 (−0.004 to 0.012)	1.10 (0.88 to 1.50)	
**Secondary effectiveness outcomes**					
Death prior to hospital discharge – no. /total no. available (%)	48/4,216 (1.1)	47/4,230 (1.1)	0.001 (−0.005 to 0.005)	1.0 (0.69 to 1.50)	
Death within 90 days – no./total no. available (%)^e^	90/3,857 (2.3)	83/3,867 (2.1)	0.002 (−0.005 to 0.008)	1.10 (0.80 to 1.40)	
Hospital length of stay – median [IQR]	5.0 [3.0, 9.0]	5.0 [3.0, 9.0]		0 (0 to 0)	
No. available	4,211	4,226			
Hospital-free days out of 28 days – median [IQR]	23 [19, 25]	23 [19, 25]		0 (0 to 0)	
No. available	4,209	4,222			
**Safety outcomes** ^f^					
Thrombosis – no./total no. available (%)	55/4,216 (1.3)	55/4,230 (1.3)	0 (−0.005 to 0.005)	1.00 (0.69 to 1.50)	0.91
Cerebral edema – no./total no. available (%)	18/4,216 (0.43)	17/4,230 (0.40)	0.001 (−0.003 to 0.003)	1.10 (0.55 to 2.10)	0.57
Sodium >155 mEq/L – no./total no. available (%)	52/2,805 (1.9)	89/2,862 (3.1)	−0.013 (−0.021 to −0.005)^g^	0.60 (0.43 to 0.84)^g^	0.003
Sodium <128 mEq/L – no./total no. available (%)	2/2,805 (0.07)	0/2,862 (0)	---	---	---
Chloride >110 mEq/L – no./total no. available (%)	866/2,751 (31)	1,378/2,813 (49)	−0.18 (−0.20 to −0.15)^g^	0.64 (0.60 to 0.69)^g^	<0.001
Potassium >6 mEq/L – no./total no. available (%)	63/2,773 (2.3)	73/2,834 (2.6)	−0.003 (−0.01 to 0.005)^g^	0.88 (0.63 to 1.20)^g^	0.45
Calcium total >12 mg/dL or ionized >1.35 mmol/L – no./total no. available (%)	125/919 (14)	139/1,001 (14)	−0.0028 (−0.03 to 0.03)^g^	0.94 (0.76 to 1.20)^g^	0.60
Lactate >4 mmol/L – no./total no. available (%)	259/1,296 (20)	228/1,348 (17)	0.03 (0.001 to 0.06)^g^	1.20 (1.00 to 1.40)^g^	0.04

a Outcomes are reported from the number available with observed (i.e., not missing) data

b The risk difference is reported as the difference in proportions from the multiple imputed datasets and combined using Rubin’s rules

c The effect measure is reported as site-adjusted risk ratios with 95% confidence intervals estimated using the Mantel–Haenszel method and combined across multiple imputed datasets, or as median differences with 95% confidence intervals estimated using the Hodges–Lehmann estimator and combined across multiple imputed datasets

d P-values are from the Cochran–Mantel–Haenszel test for binary outcomes stratified by study site and from the Van Elteren test for continuous outcomes stratified by study site, without adjustment for multiple comparisons for the primary and safety outcomes

e Death within 90 days was missing if data were not available in the study site medical record

f Safety outcomes must have occurred within 4 calendar days of randomization, except thrombosis which must have occurred within 7 days of randomization

g The risk difference and risk ratio is reported only from patients who had measured values between 2 hours after randomization (on study day 0) through study day 3
