# Supplementary material for: Balanced Fluid versus 0.9% Saline in Children Treated for Septic Shock
Source: N Engl J Med. Author manuscript; Available in PMC 2026 May 1. (PMC13134814; doi:10.1056/NEJMoa2601969)
Supplement: supplment [file NIHMS2161327-supplement-supplment.pdf]

Balanced Fluid versus 0.9% Saline in Children Treated for Septic Shock

Supplementary Appendix

Contents

PRoMPT BOLUS INVESTIGATORS ……………………………………………………………...4

SUPPLEMENTAL METHODS…………………………………………………………………..10

A. Trial Registration and Human Subjects Oversight………..…………………………………10

B. Informed Consent……………………………………………………………………………..11

C. Eligibility Criteria…………………………………………………………...……...……14

D. Randomization and Allocation Concealment……………………………..………………17

E. Pragmatic Data Collection……………………………………………...………….……19

F. Handling of Missing Weight………………………………………………..……………21

G. Handling of Missing Baseline Creatinine………………………………..………………...23

H. Definitions of Effectiveness and Safety Outcomes…………………………..……….……25

I. Handling of Missing Outcome Data Using Multiple Imputation…………………….…...…….28

J. Criteria for Early Termination of Study Fluid Administration…………………….…….……33

K. Monitoring for Protocol Adherence……………………………………………….…………34

L. Interim Analyses……………………………………………………….………..…….35

M. Tipping Point Analysis and Additional Sensitivity Analyses…………………….………..37

N. Subgroup Analyses……………………………………………………………….………...40

O. Determination of Adverse Events………………………………………………...……..42

SUPPLEMENTAL FIGURES……………………………………………….……………….………43

Figure S1. Study Overview…………………………………………..……………….……..43

Figure S2. Projected and Actual Participant Enrollment……………………….…….………...44

Figure S3. Screening, Randomization, and Analysis…………………………….………..…..….45

Figure S4. Type of Informed Consent……………………..……………..…….………………47

Figure S5: Fluid Volume by Study Phase……………………….………..…………..……..48

Figure S6. Components of the MAKE30 Composite Outcome……….….………….….….…49

Figure S7: Results of the Tipping Point Analysis for MAKE30……………….………………....50

Figure S8. Mortality Within 90 Days……………………………….……..………..….........51

Figure S9. Box Plot of Laboratory Values by Treatment Arm……………………..……….….52

Figure S10. Results of the Tipping Point Analysis for Safety Outcomes…………………...........53

Figure S11. Chloride Concentration Relative to Volume of Crystalloid…………………............54

Figure S12. Total CO2 (Bicarbonate) Concentration Relative to Volume of Crystalloid…………55

Figure S13. Serum Creatinine Relative to Volume of Crystalloid…………………………………56

Figure S14: Subgroup Analysis of MAKE30 by Measured versus Imputed Baseline Serum Creatinine…………………………………………………………………………………………57

Figure S15. Additional Subgroup Analyses of MAKE30 by Initial Illness Severity………………58

Figure S16. Heterogeneity of Treatment Effect of MAKE30 by Fluid Volume……..……..……….59

SUPPLEMENTAL TABLES……………………………………………………………………...60

Table S1. Composition of Human Plasma and Crystalloid Fluids……………………….............60

Table S2. Study Procedures and Timeline……………………………………………….............61

Table S3. Detailed Reasons for Not Eligible and Missed Eligible Patients….…………………….63

Table S4. Country of Enrollment………………………………………………………………65

Table S5. Additional Demographic Characteristics of Participants of Participants………………....66

Table S6. Site of Infection…………………………………………………………………………...68

Table S7. Alternative Diagnoses for Participants Found Not to Have Sepsis………………………69

Table S8. KDIGO AKI Stage by Measured or Imputed Baseline Serum Creatinine……………....72

Table S9. Treatments for Sepsis from Presentation Through End of Intervention Phase………….73

Table S10: Fluid Administration……………………………………………….………..…….74

Table S11. Sensitivity Analyses for Effectiveness Outcomes……..……………………..………77

Table S12. Sensitivity Analyses for Pre-randomization 0.9% Saline Administration…..…………..79

Table S13: Median and Range of Laboratory Values by Treatment Arm…………….…………80

Table S14. Sensitivity Analyses for Safety Outcomes…………………………….……………..82

Table S15. Adverse Events……………………………………………………….……….........83

Table S16. Representativeness of Study Participants………………………….………………85

SUPPLEMENTAL REFERENCES……………………………………….……….…..……….88

PRoMPT BOLUS Investigators

International Steering Committee:

Study Principal Investigators: Fran Balamuth, MD PhD MSCE (USA); Scott L. Weiss, MD MSCE (USA)

Program Managers: Ruchi Singh, PhD (USA); Amanda Williams, BN (Australia/New Zealand); Beata Mickiewicz, PhD (Canada)

Data Coordinator: Atzael B. Campos, MS (USA)

Biostatisticians: Amanda Artis, MS, MPH (USA); Jing Huang, PhD (USA)

Network Principal Investigators: Graham C. Thompson, MD (Canada); Elliot Long, BMBS PhD (Australia); Meredith Borland, MBBS (Australia); Stuart R. Dalziel, MBChB PhD (New Zealand); Adriana Yock-Corrales, MD, MSc (Costa Rica)

Senior Network Leads: Stephen B. Freedman, MDCM MSc (Canada); Franz E. Babl, MD MPH DMedSc (Australia/New Zealand)

Study Senior Investigator: Nathan Kuppermann, MD MPH (USA)

Program Administrator: Christopher P. Hickey, BA

Trial Co-Investigators:

Nephrology Consultant: Benjamin L. Laskin, MD MS

Critical Care Nephrology Consultant: Julie Fitzgerald, MD PhD MSCE

<u>Site Investigators and Co-Investigators</u>:

Country	Site	Site-PI (ED)	Site Co-I (PICU)	Other Study Team Members
USA	Children's Hospital of Philadelphia, Philadelphia, PA	Fran Balamuth MD, PhD MSCE; Rebecca Green, MD	Julie C. Fitzgerald, MD PhD MSCE	S. Arruda<br>E. Kierian<br>K. Lau<br>E. Tsemberis<br>A.Vohra
USA	Children's Hospital of Pittsburgh, Pittsburgh, PA	Robert W. Hickey, MD	Joe Carcillo, MD	M. Dean<br>S. Lorence<br>S. Young<br>A. Zimmerman
USA	University of California at Davis Children's Hospital, Sacramento, CA	Cheryl Vance, MD	Moonjoo Han, MD	A. Andrade<br>R. Dines<br>S. Graham<br>N. Okawa<br>K. Pimenta
USA	C.S. Mott Children's Hospital, Ann Arbor, MI	Alexander Rogers, MD	Stephen Gorga, MD	V. Cervantes<br>P. Chahal<br>K. Foley<br>S. Schick
USA	Nationwide Children's Hospital, Columbus, OH	Julia Lloyd, MD	Mark Hall, MD	K. Clinton<br>R.Czarnecki<br>B. Lang<br>L.Lee<br>D. Nelson<br>K. Rengel<br>B. Vance
USA	Cincinnati Children's Hospital Medical Center, Cincinnati, OH	Michelle Eckerle, MD	Erika Stalets, MD	N. Gamel<br>V. Kahman<br>M. Singleton<br>M. Schloemer

USA	St Louis Children's Hospital, St. Louis, MO	Lindsay D. Clukies MD	Matthew Goldsmith MD	D. Robinson
USA	Children's Wisconsin, Milwaukee, WI	Shannon Baumer-Mouradian, MD	Nathan Thompson, MD	M. Armanious<br>A. Radowicz<br>E. Rice<br>S. Torres
USA	New York Presbyterian Morgan Stanley Children's Hospital, New York, NY	Maria Y. Kwok, MD MPH	Patrick Wilson, MD, MPH	T. Adler<br>V. De La Cruz<br>J. Ochs<br>M.I. Ne<br>S. Petttway<br>Y. Toribio
USA	Texas Children's Hospital, Houston, TX	Julie K. McManemy, MD MPH	Trung Nguyen, MD	V. Gonzales
USA	Children's Hospital Colorado, Aurora, CO	Lilliam Ambroggio, PhD MPH	Erin Stenson, MD	A.Edid<br>R. Helmuth<br>A. Islas<br>J. Jordan
USA	Seattle Children's Hospital, Seattle, WA	Neil G. Uspal, MD	Jerry Zimmerman, MD	R. Kwon<br>C. Nguyen<br>C. Smith<br>B. Strelitz<br>M. Tosch-Berneburg<br>C. Tran<br>R. Wilbur
USA	University of California San Francisco, Benioff Children's Hospital, San Francisco, CA	Karim Mansour, MD	Natalie Cvijanovich, MD	L. Lavrisha<br>K. Li
USA	Children's Hospital Los Angeles, Los Angeles, CA	Ara Festekjian, MD MS	Fernando Beltramo, MD	J. Perez<br>S. Ringold<br>G. Sant'Ana
USA	Emory Children's Healthcare of Atlanta, Atlanta, GA	Claudia Morris, MD	Jocelyn Grunwell, MD	L. Covelo<br>A. Hoyos<br>M. McCloskey

				M. Yasmine
USA	Hasbro Children’s, Providence, RI	Susan Duffy, MD	Ranna Rozenfeld, MD	I. Bahamon<br>N. Hinz<br>M. Johnbaptiste<br>A. Zahra
USA	Children’s Medical Center of Dallas, Dallas, TX	Mohamed Badawy, MD	Archana Dhar, MD	A.Bayo<br>J. Dodson<br>M. Hamilton<br>B. Paul<br>J. Yang
USA	Primary Children’s Hospital, Salt Lake City, UT	Roni D. Lane, MD	Jennifer Workman, MD	R. Cobes<br>T. Harbour<br>J. Jung<br>V. Villalobos
USA	Children's National Medical Center, Washington, DC	Ioannis Koutroulis, MD PhD MBA	Michael Bell, MD	S.Latimer<br>M. Walker
USA	Boston Children's Hospital, Boston, MA	Matthew A. Eisenberg MD MPH	Daniel Kelly, MD; Kate Madden, MD	R. Aresco<br>C. Drescher<br>M. Du
USA	Ann and Robert H. Lurie Children's Hospital of Chicago, Chicago, IL	Elizabeth Alpern, MD MSCE; Priya G. Jain, MD MEd	Matthew Barhight, MD	J. Benedetti<br>J. Kapes<br>K. Lehnig<br>L. McDevitt<br>M.Samuels<br>R. Shannon<br>A.Sirizi<br>R.Turrick
USA	Children’s Hospital of Richmond at Virginia Commonwealth University, Richmond, VA	Jonathan Silverman, MD	Nikki Miller Ferguson, MD	M. Scott
Canada	Alberta Children's Hospital, Calgary, AB	Graham Thompson, MD	Suzette Cooke, MD PhD; Elyahueli Gilad, MD	B. Mickiewicz

Canada	British Columbia Children's Hospital, Vancouver, BC	Pavan Judge, MBBS	Srinivas Murthy, MD	K. Stillwell<br>N. Kissoon
Canada	Children's Hospital of Eastern Ontario, Ottawa, ON	Waleed Alqurashi, MD MSc; Fuad Alnaji MD	---	C. Goodkey
Canada	London Health Sciences Centre, London, ON	Gary I. Joubert, MD	---	P. Siedlecki
Canada	Stollery Children's Hospital, Edmonton, AB	Sarah J. Curtis, MD MSc	Ari Joffee, MD	R. Odsen
Canada	Centre Hospitalier Universitaire Sainte Justine, Montréal, QC	Yasaman Shayan, MDCM MS-HPEd	Marisa Tucci, MD	R. Cook<br>F. Drapeau-Jacob
Canada	HSC Winnipeg Children's Hospital, Winnipeg, MB	Karen E. Gripp, MD	---	A. Bellemare
Canada	Centre Hospitalier de l'Université Laval, Québec City, QC	Simon Berthelot, MD	Matthew J. Weiss, MD	H. Kermiche<br>N. Boudaa
Canada	Hospital for Sick Children, Toronto, ON	Adrienne L. Davis, MD MSc	Elaine Gilfoyle, MD	T. Agarwal<br>B. Lerman<br>M. Moretti
Canada	McMaster Children's Hospital, Hamilton, ON	April Kam, MD MPH	Melissa Parker, MD MSc	R. Carciumaru<br>B. Rochwerg
Canada	IWK Health Centre, Halifax, NS	Jason G. Emsley, MD MSc PhD	Neeraj Verma, MBBS DNB	N. McCaughey<br>K. Trask
Canada	Kingston Health Sciences Centre, Kingston, ON	Anupam Sehgal, MBBS DNB	Anne Moffatt, MD	A.Hui
AUST	The Royal Children's Hospital, Melbourne, VIC	Elliot Long, BMBS PhD; Franz Babl MD MPH DMedSc	Ben Gelbart, PhD MBBS; Warwick Butt, MBBS	A.Williams<br>S.McNab
AUST	Women's and Children's Hospital, Adelaide SA	Amit Kochar, MBBS MD MPH	Subodh Ganu, MBBS MD	G.Nievea

AUST	The Royal Darwin Hospital Tiwi, NT	Anna Lithgow, MBBS MPH	---	M. Duck
AUST	Sydney Children’s Hospital, Randwick, NSW	Arjun Rao, MBBS MAppSci	Puneet Singh, MBBS MD	C. Baldock
AUST	Perth Children’s Hospital, Nedlands, WA	Meredith Borland, MBBS	Simon Erickson, MBBC DipECHO	S. O’Brien
AUST	Queensland Children’s Hospital, South Brisbane, QLD	Natalie Phillips, MBBS MPhil	Sainath Raman, MBBS PhD	A.Jones
AUST	Gold Coast University Hospital, Southport, QLD	Shane George, MBBS PhD	---	K. Owen
AUST	The Children’s Hospital at Westmead, Westmead NSW	Shefali Jani, MD	Marino Festa, MD	D. Thosar
AUST	Townsville University Hospital, Douglas, QLD	Emma Whyte, MBBS	---	C. West
AUST	Monash Medical Centre, Clayton, VIC	Simon Craig, MBBS, MPH MHPE PhD	Felix Oberender, MRCPCH PhD	C. Le
NZ	Middlemore Hospital, Auckland, NZ	Eunicia Tan, MBChB	---	A. Grigg
NZ	Starship Children’s Hospital, Auckland, NZ	Stuart Dalziel, MBChB PhD	Anusha Ganeshalingham, MBChB	M. Rao
Costa Rica	Hospital Nacional de Niños “Dr. Carlos Sáenz Herrera”, San Jose	Adriana Yock-Corrales, MD MSc	David Navarro-Salas, MD	

A. Trial Registration and Human Subjects Oversight

The Pragmatic Pediatric Trial of Balanced Versus Normal Saline Fluid in Sepsis (PRoMPT BOLUS) was written as four protocols to conform to human subjects regulations specified within the United States (US), Canada, Australia/New Zealand, and Costa Rica. However, the study was registered as a single trial in clinicaltrials.gov (NCT04102371) and was analyzed as a single trial as pre-specified in the analysis plan published in *Trials* prior to concluding enrollment. The study was designed and the manuscript was written in accordance with the CONSORT recommendations for pragmatic clinical trials.[1]

A single Institutional Review Board at the Children's Hospital of Philadelphia (CHOP) provided regulatory oversight for all US sites. The University of Calgary received regulatory authorization from Health Canada. In Canadian provinces hosting provincial Research Ethics Board (REB) programs (i.e., Alberta, Ontario, Québec), the lead sites received initial approval, following by a streamlined review and approval process at the remaining sites in those provinces. All remaining Canadian sites received REB approval from their specific institutions. A single Research Ethics Committee at the Royal Children's Hospital, Parkvile provided regulatory oversight for Australian sites, and the Northern A. Health and Disability Ethics Committee provided regulatory oversight for New Zealand sites. In Costa Rica, the Hospital Nacional de Niños Research Ethics Board approved the study.

B. Informed Consent

Due to the life-threatening nature of septic shock and narrow therapeutic window to commence fluid resuscitation, alterations to prospective written informed consent were used for this study. Such enrollment methods varied slightly according to the regulations in place within individual countries and at the direction of local review boards, but all alterations were determined to be ethically suitable for emergent, life-threatening conditions when patients may benefit from the research, available treatments are unproven/unsatisfactory, and obtaining prospective informed consent is not feasible.[2-6] One site in the United States (US) was ultimately unable to participate due to local concerns noted during consultation with community members.

In the US, enrollment adhered to "Exception From Informed Consent" (EFIC) under 21 CFR 50.24 for emergency research.[7] Per federal guidelines for studies approved using EFIC, the study protocol was approved under an Investigational New Drug (IND #136978) application by the US Food and Drug Administration (FDA) with annual progress reports submitted. As per regulatory requirements for EFIC, all US sites completed community consultation and public disclosure activities (available at https://www.regulations.gov/docket/FDA-1995-S-0036) to obtain input from and inform the local community prior to beginning enrollment.[8,9] Beginning prior to enrollment, potential participants could choose to "opt-out" of future enrollment by registering on a website or verbally indicating their preference not to be enrolled in the study. A centralized opt-out list was maintained throughout the study and was distributed to all US sites each time a patient was added to this list at any US sites. When time permitted, prospective written informed consent was offered to the patient's legally authorized representative (LAR). If there was not sufficient time for a full informed consent discussion prior to enrollment, but the LAR was present and immediately accessible, then a brief verbal introduction to the study took

place to allow for the LAR to “opt-out”. Otherwise, patients confirmed to meet all eligibility criteria were enrolled under EFIC with randomization to a treatment group and initiation of study procedures.

In Australia, a process for a waiver of the requirement for consent to begin study procedures followed by delayed consent to continue in the study was approved in compliance with the National Statement on Ethical Conduct in Human Research.[10] If the patient remained seriously unwell or died, a waiver for consent to continue was obtained to continue collection of data and include thee patients in the analysis. For patients enrolled in Western Australia, prospective informed consent was preferred but if this was not feasible, randomization proceeded under Independent Medical Practitioner (IMP) consent. LAR consent to continue in the study was sought for most patients, but if the patient remained severely unwell or died, the IMP provided full consent. In New Zealand, a process of delayed consent to continue in the study was approved. In Canada, a process for deferred consent was approved in compliance with the Canadian Tri-Council Policy Statement-2.[11] In Costa Rica, a process for deferred consent was approved by the local research ethics board of Hospital Nacional de Niños “Dr. Carlos Sáenz Herrera”.

At all sites, participants enrolled through an alteration of prospective informed consent (and the LAR) were notified of enrollment and study procedures as soon as feasible after randomization and offered the right to continue or withdraw from the study. For study participants who died prior to the post-enrollment discussion, reasonable attempts were made to inform the LAR after the participant’s death, unless a waiver was granted in local jurisdictions. However, successful LAR contact was not required to for a participant’s data to be included in the analyses.

The requirement for assent was waived at US sites, but assent sought from patients who were deemed to have capacity in Canada, New Zealand, and Costa Rica. In Australia, patient consent was sought from those deemed to have capacity.

C. Eligibility Criteria

Patients >2 months to <18 years of age treated for suspected septic shock for whom the treating clinician planned to administer parenteral antibiotics and at least one fluid bolus for abnormal perfusion in a study site ED were eligible for study enrollment. "Suspected septic shock" was operationalized as a) the treating clinician diagnosis of septic shock and/or treatment that included a parenteral antibiotic and initiation of fluid resuscitation (or plan for more than one fluid bolus) for abnormal perfusion or b) a sepsis alert on a site-specific screening tool with clinician confirmation to proceed with treatment for suspected septic shock. Abnormal perfusion was defined as the treating clinician's judgement that hypotension or abnormal (either "flash" or "prolonged") capillary refill was present. To reflect usual clinical practice, we did not define thresholds for hypotension or abnormal capillary refill but rather deferred to the treating clinician's discretion to differentiate abnormal from normal. Recognizing that fluid resuscitation may start prior to ED arrival, only those for whom total volume of crystalloid fluid administration was confirmed as ≤40 mL/kg prior to enrollment were eligible. Enrolled patients who were discharged from the hospital were eligible to participate again if they were re-presented to a study hospital with suspected septic shock.

The lower age limit for enrollment was initially set at >6 months to adhere to the United States Food and Drug Administration (FDA) warning that infants <6 months may have reduced hepatic capacity to metabolize exogenous lactate in lactated Ringer's, but was lowered to >2 months on February 1, 2023, after further evidence of safe use of balanced fluid in infants 2-6 months of age[12] and the applicability of Surviving Sepsis Campaign conditional recommendation to use balanced fluids in pediatric septic shock to this age group.[13]

Exclusion criteria were 1) prior indication that the patient would not agree to be enrolled in the study, 2) clinician judgement that the patient's condition deemed it unsafe to administer

either 0.9% saline or balanced fluids, including (but not limited to) preexisting chronic kidney disease (CKD) requiring maintenance renal replacement therapy, fulminant acute liver failure (defined as defined as blood alanine aminotransferase [ALT] >10,000 U/L or total bilirubin >12.0 mg/dL), or known metabolic disorder, inborn error of metabolism, or primary mineralocorticoid deficiency (see below for additional detail), 3) receipt of >40 mL/kg crystalloid fluid before randomization (or inability to confirm prior fluid volume received), 4) known pregnancy, 5) known prisoner status, or 6) known allergy to either fluid type. Patients under child protective services custody were excluded at some sites in compliance with local regulations. Although not a pre-specified exclusion criterion, some patients were also excluded for a perceived language barrier. Due to the pragmatic nature of the study embedded within clinical practice, the reason for lack of eligibility was not recorded for all patients who were screened.

Although final assessment of safe use of either fluid type was left to the discretion of the treating clinician, the following conditions—if known at time of assessment for eligibility—were suggested reasons for exclusion based on unclear safety of either 0.9% saline or balanced fluids:

- Suspicion for impending brain herniation
- Known hyperkalemia (serum or whole blood potassium >6 mEq/L)
- Known hypercalcemia (total calcium >12 mg/dL or ionized calcium > 1.35 mmol/L)
- Known acute fulminant hepatic failure (alanine aminotransferase >10,000 U/L or total bilirubin >12.0 mg/dL)
- Known history of severe hepatic impairment, defined as diagnosis of cirrhosis or liver failure, or active listing for liver transplant

- Known history of severe kidney disease, defined as current dependency on peritoneal dialysis or hemodialysis
- Known metabolic disorder, inborn error of metabolism, or primary mineralocorticoid deficiency (e.g., mitochondrial disorder, urea cycle disorder, amino acidemia, fatty acid oxidation disorder, glycogen storage disorder, congenital adrenal hypoplasia, Addison's disease)

Screening for missed eligible patients was performed either continuously or intermittently at study sites. At a minimum, sites screened for at least one week each quarter and identified patients treated for septic shock who were not enrolled. Sites with established electronic sepsis protocols were able to screen for missed eligible patients continuously throughout the enrollment period. The proportion of enrolled eligible patients was tracked for each site and reported back to each site at least quarterly. Sites that enrolled less than 80% of eligible patients received targeted education and more frequent tracking.

D. Randomization and Allocation Concealment

We used patient-level, rather than clustered, randomization to reduce risk of selection bias, limit provider confusion over the recommended fluid type, and avoid exposing non-study patients to the intervention. Patients were randomized to receive either 0.9% saline or balanced fluids. Equal allocation randomization tables were prepared by the Data Coordinating Center using permuted block randomization stratified by clinical site. Permuted blocks of random lengths were used to minimize bias due to the potential of predictable assignments with fixed block sizes. Treatment assignments were placed into sequentially numbered opaque envelopes and stored at each site in close proximity to the crystalloid fluids. Specifically, for each clinical center, sequences were prepared as follows:

1. Block length was selected randomly (length 2, 4, or 6, with equal probability)
2. For the selected block length, the treatment sequence was randomly shuffled, and the resulting sequence of treatments added to the existing randomization sequence for the center stratum
3. The process above was repeated until a list of size 1,000 was generated for each site.

Randomization was performed in the R statistical package. Block length was randomly selected and treatment assignments were randomly shuffled within blocks using the sample() function. Randomization seeds were selected and recorded to enable reproducibility of treatment sequences if necessary.

Randomization sequences were delivered in batches to each study site in either pre-sealed envelopes or as confidential electronic lists to be locally placed in sealed envelopes by staff who were not involved with determining patient enrollment. Study group allocation was then concealed in the serially numbered, opaque envelopes, which provided an efficient process for

quickly randomizing participants at the bedside concurrent with ongoing clinical management. Treatment allocation to 0.9% saline or balanced/buffered fluids was revealed after eligibility for enrollment has been confirmed. The randomization/envelope number was recorded in the database. Any patient for whom an envelope was opened was considered to have been enrolled and randomized.

Study participants and clinicians were not blinded to treatment allocation. Attempting to blind study fluid was deemed potentially unsafe for patients and detrimental to protocol adherence, as clinicians may have incorrectly attributed physiologic and/or biochemical changes to study fluid type, prompting unnecessary use of non-study fluid. Moreover, it is unlikely that blinding would have been possible because available laboratory values can overtly reflect the fluid type being used.[14] However, the senior biostatistician (JH) and all investigators remained blinded to aggregate outcomes until after enrollment was completed.

Finally, we note that the study intervention applied only to bolus fluids and the base fluid used for maintenance hydration. Due to practical challenges of medication preparation and safety concerns about medical compatibility with certain fluids, crystalloid fluids used for diluents and medication "carriers" were not included in the intervention.

E. Pragmatic Data Collection

Data were extracted from medical records and recorded on standardized case report forms. Each network (i.e., PECARN, PERC, PREDICT, and Costa Rica) supervised data collection and quality from its respective sites. In the US, data were collected through the FDA-compliant Advarra Electronic Data Capture (Columbia, MD) hosted at the Children's Hospital of Philadelphia. In Canada, Australia/New Zealand, and Costa Rica, data were collected in Research Electronic Data Capture (REDCap, Vanderbilt University; Nashville, TN) hosted at the University of Alberta, the Murdoch Children's Research Institute, and Hospital Nacional de Niños Dr. Carlos Saenza Herrera, respectively. However, all sites collected the same data elements using a common data dictionary. All study data were password-protected and coded with a study number to ensure confidentiality. Study personnel at each site were trained to collect data using two test cases and accuracy of data extraction was compared to source documents at least annually within each network to certify data accuracy. The US-based Data Coordinating Center (DCC) located at the Children's Hospital of Philadelphia centrally managed and collated all data exported from Advarra and REDCap to ensure harmonization for analyses. Consistent with the tenets of a pragmatic trial, data collection was purposely brief and targeted key patient characteristics, fluid administration, and outcomes, as listed below:

Category	Data Elements Collected
Demographics	Age, race, ethnicity, sex, weight, comorbid conditions
Hospital summary	Admission disposition, length of stay, discharge disposition
Fluid administration	Crystalloid and colloid fluid volume, timing, and composition, blood product administration, parenteral nutrition collected from presentation

	to the emergency department through study day 2 (i.e., the end of the intervention phase)
Laboratory data	Initial and follow-up blood electrolytes, lactate, total carbon dioxide concentration (“bicarbonate”), creatinine, alanine aminotransferase (ALT), baseline creatinine; follow-up laboratory data was collected through study day 3 (i.e., 24 hours after the end of the intervention phase) if measured as part of clinical care
Microbiology	Site of infection, pathogen(s), bacteremia
Therapies	Antibiotics (type, timing), vasoactive medications, bicarbonate/acetate (or other acid buffers), corticosteroids, mechanical ventilation, extracorporeal membrane oxygenation that occurred through the end of the intervention phase
Outcomes	Blood creatinine at discharge or 30 days post-enrollment (whichever came first), inpatient renal replacement therapy, vital status at hospital discharge and 90 days post-enrollment, arterial/venous thromboembolism, cerebral/brainstem herniation
Adverse events	A select list of untoward medical occurrences in a study participant through the end of study day 7

F. Handling of Missing Weight

Weight was used to standardize volumes of fluid received into a common unit of mL/kg. If body weight was not measured or available, the median weight for age and sex based on the United States Centers for Disease Control and Prevention recommendations was used for fluid volume calculations.[15]

Age	Male (kg)	Female (kg)
2 months	5.6	5.1
3 months	6.4	5.8
4 months	7.0	6.4
5 months	7.5	6.9
6 months	7.9	7.3
7 months	8.3	7.6
8 months	8.6	8.0
9 months	8.9	8.3
10 months	9.2	8.6
11 months	9.4	8.9
12 months	9.6	9.2
2 years	12.2	11.5
3 years	14.3	13.7
4 years	16.1	15.6
5 years	17.8	17.3
6 years	19.5	19.0

7 years	21.3	20.8
8 years	23.2	22.6
9 years	25.4	24.0
10 years	32.1	33.1
11 years	36.0	38.3
12 years	41.1	43.5
13 years	45.9	47.5
14 years	50.1	52.2
15 years	54.1	55.9
16 years	57.2	57.8
17 years	59.5	58.7
18 years	60.5	59.0

G. Handling of Missing Baseline Creatinine

For participants for whom serum creatinine was not available between 12 months and 24 hours prior to enrollment, an imputed value using established median values for age and sex was used to define baseline serum creatinine.[16] In a prior publication, we demonstrated that this approach resulted in a conservatively imputed value for serum creatinine at or above the upper limit of the 95% confidence limit for age and sex compared to measured baseline serum creatinine from 6 months to 18 years-old.[16] We also previously demonstrated that alternative approaches to impute baseline serum creatinine when it was not available between 12 months and 24 hours prior to ED presentation had little impact on the incidence of MAKE30.[16] Thus, we used the median creatinine values indicated in the table below to determine baseline kidney function for patients without measured creatinine:

Age	Median Serum Creatinine (mg/dL)
2 mos to <1 year	0.25
1 to < 2 years	0.27
2 to <3 years	0.30
3 to <4 years	0.33
4 to <5 years	0.36
5 to <6 years	0.38
6 to < 7 years	0.43
7 to < 8 years	0.45
8 to <9 years	0.47
9 to <10 years	0.50
10 to <11 years	0.52
11 to <12 years	0.54
12 to <13 years	0.57
13 to <14 years	0.61

	Male	Female
14 to <15 years	0.68	0.62
15 to < 16 years	0.78	0.68
16 to <17 years	0.82	0.70
17 to <18 years	0.85	0.71

H. Definitions of Effectiveness and Safety Outcomes

The primary outcome was the occurrence of a **major adverse kidney event at 30 days (MAKE30)** after study enrollment or hospital discharge, whichever occurred first. MAKE30 was defined as at least one of the following:

- **Death** (from any cause) *or*
- **New renal replacement therapy** (or attempt to treat if not tolerated) *or*
- **Persistent kidney dysfunction**, defined as serum creatinine ≥2x baseline or median value for age if no baseline available *and* a minimum absolute increase in serum creatinine of ≥0.3 mg/dL from baseline)

An occurrence of MAKE30 was defined if at least one of the components was known to occur but was only defined as not occurring if all three components were observed to be negative (e.g., if patient was known to survive to hospital discharge and was not treated with renal replacement therapy, but did not have a qualifying serum creatinine measured prior to hospital discharge, MAKE30 was defined as "missing").

The rationale for MAKE30 as the primary endpoint was that mediation of kidney injury provides the major mechanistic pathway through which balanced fluids are likely to improve patient outcomes. In addition, the only prior comparable randomized clinical trials of balanced fluids at the time of study design and implementation similarly focused on MAKE30 or similar kidney-focused outcomes.[17,18] Finally, MAKE30 provided an objective and easily measured outcome endorsed as a patient-centered endpoint for clinical trials.[19]

Secondary effectiveness outcomes were the components of MAKE30 and the following:

- **Death prior to hospital discharge**, defined has all-cause mortality at hospital discharge

- **Death within 90 days**, defined as all-cause mortality at 90 days using data available from the medical record indicating either date of death or evidence of life after day 90 (e.g., documentation of contact with the health system after 90 days from randomization)
  Note: Mortality was determined only from the medical record as data from the US National Death Index and Canadian provincial vital statistics, as originally planned, were not able to be ascertained
- **Hospital length of stay**, defined as the number of days from hospital admission until discharge, censored at 90 days
- **Hospital-free days out of 28 days**, defined as the number of days between enrollment and day 28 in which the patient was both alive and out of the hospital. Patients who died prior to hospital discharge were recorded as having "zero" hospital-free days.

Safety outcomes were monitored based on data available through the course of usual clinical care and were defined as the following:

- **Hyperlactatemia**, defined as at least one arterial or venous blood lactate measurement >4 mmol/L within 4 calendar days of randomization
- **Hyperkalemia**, defined as at least one venous, arterial, or capillary blood potassium measurement >6 mEq/L within 4 calendar days of randomization
- **Hypercalcemia**, defined as at least one venous, arterial, or capillary blood ionized calcium measurement >1.35 mmol/L or total venous, arterial, or capillary blood calcium measurement >12 mg/dL within 4 calendar days of randomization
- **Hypernatremia**, defined as at least one venous, arterial, or capillary blood sodium measurement >155 mEq/L within 4 calendar days of randomization

- **Hyponatremia**, defined as at least one venous, arterial, or capillary blood sodium measurement <128 mEq/L within 4 calendar days of randomization
- **Hyperchloremia**, defined as at least one venous, arterial, or capillary blood chloride measurement >110 mEq/L within 4 calendar days of randomization
- **Thrombosis**, defined as either a) treatment for new arterial or venous thrombus with systemic anticoagulant or b) clotting of intravenous central catheter in patients receiving ceftriaxone and lactated Ringer's within 7 days of randomization (thromboembolism was monitored for 7 days since diagnosis of this event could have been delayed beyond the inciting event)
- **Cerebral edema**, defined as treatment with hyperosmolar therapy (i.e., hypertonic saline and/or mannitol) for radiographic and clinical determination of new impending or present brain herniation within 4 calendar days of randomization (so long as a clinical diagnosis of brain herniation is not disproven by radiographic studies)

I. Handling of Missing Outcome Data Using Multiple Imputation

Framework

We used multiple imputation by fully conditional specifications (FCS; also known as multivariate imputation by chained equations) to address missing data in outcomes. Imputation was conducted at the individual participant level using a single imputation model that included baseline covariates, treatment assignment, study site, and outcomes to preserve correlations among variables and ensure coherence across analyses. Imputed data sets were generated in SAS under a missing-at-random assumption conditional on observed covariates, treatment assignment, and study site (details below). Twenty imputed datasets were generated. Each imputed dataset was analyzed separately, and estimates were combined using Rubin's rules.

Variables in the Imputation Model

Variables were assigned to one of three roles in the imputation model:

1. Design variables (predictors only; not imputed):

   These were fully observed by design and included as predictors for all other variables.

   - Treatment assignment
   - Randomization site
2. Post-randomization clinical variables (used as predictors; not imputed):
   - Total crystalloid volume category
3. Baseline variables (imputed if missing):
   - These included demographic characteristics and pre-randomization clinical characteristics.
4. Outcomes (imputed if missing):

- Outcomes and their components were included in the imputation model to improve plausibility of the MAR assumption and to preserve relationships among related endpoints.

Handling of the Primary Composite Outcome (MAKE30)

The primary outcome, major adverse kidney events within 30 days (MAKE30), was defined as the occurrence of any of the following:

- death within 30 days,
- new inpatient renal replacement therapy, or
- persistent kidney dysfunction at hospital discharge.

To ensure logical consistency, the three components were imputed directly (if missing), and MAKE30 was derived deterministically from these components in each imputed dataset. MAKE30 itself was not freely imputed.

Imputation Models and Implementation in SAS

Imputation was conducted using PROC MI with the FCS statement. Variable types were handled as follows:

- Continuous variables: imputed with linear regression (FCS REG)
- Binary variables: imputed with logistic regression (FCS LOGISTIC)
- Nominal categorical variables (e.g., country, race/ethnicity, primary site): imputed using discriminant-function methods (FCS DISCRIM with class effects)
- Ordinal variables (e.g., AKI stage, total bolus volume category): imputed using discriminant-function methods as specified in the FCS DISCRIM statement.

All variables listed above were included in the VAR statement of PROC MI and were, therefore, available as predictors in the conditional models. A fixed random seed was used to ensure reproducibility. The chained-equations procedure was run with 1000 iterations per imputation step.

Analysis after Imputation

For the primary analysis, we computed a stratified estimate using Cochran–Mantel–Haenszel (CMH) methods with stratification by site. Treatment-effect estimates and standard errors were combined across imputations using Rubin's rules.

Table of Variables Included in the Multiple Imputation Model

Role in MI model	Description	Imputed?
**Design**	Treatment assignment (0.9% saline vs balanced fluid)	No
	Randomization site	No
**Post-randomization predictors**	Total crystalloid volume category	No
**Baseline --continuous**	Age in years at randomization	Yes
	Baseline serum creatinine	Yes
	Body weight in kg	Yes
	Minutes to first antibiotic administration after ED arrival	Yes
**Baseline --binary**	Sex	Yes
	Cancer (hematogenous or solid tumor)	Yes

	Bone marrow or solid organ transplant	Yes
	Cardiomyopathy or heart failure	Yes
	Pulmonary hypertension	Yes
	Kidney disease (not on dialysis)	Yes
	Neurologic dysfunction causing severe developmental delay	Yes
	Sickle cell disease	Yes
	Chronic ventilator dependence	Yes
	Indwelling central line	Yes
	Positive blood culture (bacteremia) as either primary or additional site of infection	Yes
	Antibiotics prior to study site ED arrival	Yes
	Ceftriaxone	Yes
	Vasoactive medications	Yes
	Corticosteroids	Yes
	Bicarbonate or other buffer	Yes
	Invasive mechanical ventilation	Yes
	Extracorporeal membrane oxygenation	Yes
**Baseline -- categorical**	Country of enrollment	Yes
	Race category	Yes
	Hispanic ethnicity	Yes
	Site of infection	Yes
**Baseline -- ordinal**	Age category	No[a]

	KDIGO acute kidney injury stage at enrollment	Yes
**Primary outcome components**	Death within 30 days	Yes
	New inpatient renal replacement therapy	Yes
	Persistent kidney dysfunction at discharge (if this is the correct meaning)	Yes
**Derived composite**	Major adverse kidney events within 30 days	No[b]
**Secondary outcomes**	Death at hospital discharge	Yes
	Death at 90 days	Yes
	Hospital length of stay	Yes
	Hospital-free days out of 28	Yes
**Safety outcomes (binary)**	Thrombotic event	Yes
	Cerebral edema	Yes

[a] Age category was not imputed; missing values were derived from the imputed age in years

[b] MAKE30 was not directly imputed; instead, it was derived in each completed dataset based on the imputed values of its three component outcomes

J. Criteria for Early Termination of Study Fluid Administration

For participants for whom routine, clinician-directed physical and/or laboratory assessment revealed the following criteria within the intervention phase, clinicians were not required to continue study fluid as part of this protocol and were transitioned to an appropriate fluid as determined by the discretion of the treating clinical team.

- Hyperkalemia, defined as blood potassium >6 mEq/L confirmed on immediate repeat testing
- Hypercalcemia, defined as blood total calcium >12 mg/dL or ionized calcium >1.35 mmol/L confirmed on immediate repeat testing
- Severe hepatic impairment, defined as rise in blood ALT to >10,000 U/L or total bilirubin >12.0 mg/dL
- Severe renal impairment, defined as initiation of renal replacement therapy (continuous or intermittent hemodialysis or peritoneal dialysis) or urine output <0.5 ml/kg/hr for 16 hours
- Clinical concern for hypersensitivity to the study fluid

Forty-three participants met early termination criteria to stop study fluids. When the above early termination criteria were met, the patient remained in the study for data collection and outcome assessment. Clinician-prescribed fluid administered for remaining time in the intervention phase continued to be recorded. All 43 participants were included in the analysis, with data available for the primary outcome in 42 and imputed primary outcome used for the remaining one participant.

K. Monitoring for Protocol Adherence

Adherence to the intervention was defined as receipt of ≥75% of total crystalloid fluid as the randomized fluid type during the interventional window. For example, participants randomized to balanced fluids were considered adherent to the intervention if they received ≥75% of their total crystalloid (bolus and maintenance) as balanced fluids starting from the time of randomization through 11:59 PM on the following calendar day. Patients who did not receive any crystalloid fluid after randomization (i.e., during the intervention phase) were excluded from the per-protocol analyses and were not considered in the calculation of adherence because the protocol did not itself require that patients be treated with crystalloid fluid.

All sites were required to establish a workflow to communicate study enrollment and promote adherence across providers and hospital locations. Such strategies included verbal and/or written hand-offs between providers, use of a templated orderset indicating PRoMPT BOLUS study enrollment and treatment allocation, and automated electronic reminders to clinicians at the time an order is placed for non-study crystalloid fluids during the intervention window. Each participant's adherence to the intervention was monitored centrally, and the mean adherence to the intervention for all participants within each site was reported back to local investigators at least quarterly. Targeted education was undertaken for a site if <80% of their participants in either study arm did not meet the adherence criterion. If <70% of participants met the adherence criterion over two consecutive months, the site was placed on monitored probation with additional efforts undertaken to understand and correct challenges to adherence.

L. Interim Analyses

Interim monitoring for superiority of one treatment approach over the other was overseen during this study by a 10-panel Data Safety Monitoring Board (DSMB) with international representation and expertise in clinical trials, biostatistical analysis, pediatric sepsis, nephrology, emergency medicine, critical care, and bioethics. As both treatments are currently used as standard of care in current practice,[13] symmetric two-sided O'Brien-Fleming boundaries were used for efficacy monitoring of the main clinical endpoint of MAKE30. The significance levels at each interim analysis for difference in MAKE30 between groups were set at 0.000000014, 0.00079, and 0.014, respectively, using symmetric two-sided O'Brien-Fleming boundaries. A final significance level of 0.044 was established to be used for the final test to guarantee that the overall Type I error rate would be less than or equal to 0.05.

Interim analyses were presented to the DSMB via Microsoft Teams on June 23, 2022, June 28, 2023, and May 15, 2024 (see below). After each interim analysis, the DSMB recommended continuation of the study without modification.

Meeting Number	Study Milestone	Date
1	Pre-enrollment (USA)	July 6, 2020
2	Pre-enrollment (Canada/Australia/New Zealand)	Feb 25, 2021
3	1,192 participants enrolled (15% enrollment)	June 23, 2022
4	3,517 participants enrolled (40% enrollment)	June 28, 2023
5	6,158 participants enrolled (70% enrollment)	May 15, 2024
6	9,041 participants enrolled (100% enrollment)	January 21, 2026

The DSMB was charged with considering early termination of the trial if there was evidence of futility, defined as a limited chance of the study finding a statistically significant treatment effect with respect to the main clinical outcome (i.e., MAKE30) if the study is continued. Data were presented to the DSMB with 95% confidence intervals for the true value of the between-group treatment effect. Futility, in isolation (in absence of, for example, a safety concern), was not *a priori* determined to be sufficient to warrant stopping the trial, but the DSMB considered other factors, including the burden of finishing the trial, and the need to provide a definitive answer to the study question, in its deliberations. This approach was deemed appropriate because both 0.9% saline and balanced fluids were considered efficacious standards of care based on existing guidelines.[13] Thus, if there was truly no discernable comparative effectiveness between these two fluids, then all patients enrolled in the trial would be receiving equally efficacious therapies.

Safety endpoints and adverse events were also monitored continuously by the DCC and, at a minimum, compared between study groups at all interim analyses. Any imbalance in safety events between groups was reviewed by the DSMB at each interim analysis.

M. Tipping Point and Additional Sensitivity Analyses

We performed a pattern-mixture, delta-adjusted tipping-point sensitivity analysis to evaluate the robustness of the primary MAKE30 findings to departures from the missing-at-random (MAR) assumption. This analysis began with the multiply imputed datasets generated under MAR (20 imputed datasets). For each imputed dataset, we re-estimated a logistic regression model for MAKE30 including treatment assignment, study site, and baseline prognostic variables used in the imputation model. From this model, we obtained for each participant the linear predictor (η) and corresponding MAR-predicted probability of MAKE30.

For participants whose MAKE30 outcome was originally missing in the raw dataset, we applied an arm-specific log-odds shift (δ) to the MAR-predicted probability according to:

$$p^{(\delta)} = \text{logit}^{-1}\big(\eta + \delta \cdot I(\text{balanced fluid})\big),$$

where $I(\text{balanced fluid})$ is an indicator variable equal to 1 for the balanced-fluid group and 0 for the 0.9% saline group. Thus, for participants with missing outcomes in the 0.9% saline group, the predicted log-odds remained as estimated under MAR, whereas for those in the balanced fluid group, the predicted log-odds were shifted by δ. New outcomes for originally missing participants were drawn from a Bernoulli distribution with the predicted probability. All originally observed MAKE30 outcomes were left unchanged.

In the tipping-point analysis, δ was varied over a prespecified grid (−5 to 2). A value of δ corresponds to multiplying the odds of MAKE30 among participants with missing outcomes by e^{δ}. For example, δ = 0.7 approximately doubles the odds, and δ = 1.1 approximately triples the odds.

For each value of δ, the prespecified primary analysis (Cochran–Mantel–Haenszel method stratified by site) was re-run within each of the 20 delta-adjusted datasets. Log risk

ratios and their variances were combined using Rubin's rules to obtain the pooled treatment effect estimate, standard error, 95% confidence interval, and two-sided p-value.

The tipping point was defined as the smallest value of δ for which the pooled 95% confidence interval for the treatment effect included the null value. Results were displayed graphically as the pooled treatment effect and 95% confidence interval plotted against e^{δ}, representing the fold-increase in the odds of MAKE30 among participants with missing outcomes in the balanced fluid group.

To evaluate the robustness of the results for safety outcomes with more than 30% missing data, we conducted deterministic sensitivity analyses under two extreme assumptions, as well as a tipping point analysis using a similar approach as described above for MAKE30. For each laboratory-defined safety endpoint, treatment effects were estimated using the prespecified CMH method, stratified by study site, under two assumptions for missing data:

- *Best-case assumption (Missing = No Event)*

  All participants with missing laboratory values were assumed not to have met the safety threshold. This scenario corresponds to a conservative assumption that missingness reflects clinical stability or lack of a clinically significant abnormality (i.e., laboratory not measured by thee treating clinician because the result would be normal).

- *Worst-case assumption (Missing = Event)*

  All participants with missing laboratory values were assumed to have met the safety threshold. This represents a highly conservative scenario in which missingness is assumed to reflect unobserved safety events (e.g., laboratory abnormality existed but was not measured by the treating clinician).

For each scenario, site-adjusted risk ratios (RRs) with 95% confidence intervals were calculated as the risk in the balanced fluid group relative to the risk in the 0.9% saline group.

For the tipping-point analysis for safety outcomes, a baseline event probability was estimated separately within each treatment group using the observed data without covariate adjustment due to small number of events. For participants with missing outcomes in the 0.9% saline group, this baseline probability was used directly for imputation. For participants in the balanced fluid group, the predicted log-odds were shifted by δ to allow missing-not-at-random.

N. Subgroup Analyses

Subgroups were defined for analysis by the following stratification variables:

1. country of enrollment
    a. United States and Costa Rica
    b. Canada
    c. Australia and New Zealand
2. age group:
    a. >2 months to <1 year
    b. 1 to <5 years
    c. 5 to <12 years
    d. 12 to <18 years
3. sex
    a. male
    b. female
4. cancer
    a. yes
    b. no
5. total fluid volume (bolus and maintenance, including pre-randomization and intervention phase)
    a. <60 mL/kg
    b. 60 to 100 mL/kg
    c. >100 mL/kg
6. presence of AKI at the start of sepsis resuscitation

    a. no acute kidney injury
    b. stage 1, 2, or 3 acute injury (defined by KDIGO on serum creatinine criteria)
7. abnormal kidney function at presentation defined by KDIGO (based on serum creatinine)
    a. no acute kidney injury
    b. stage 1 acute kidney injury
    c. stage 2 acute kidney injury
    d. stable 3 acute kidney injury

No AKI was defined as serum creatinine at presentation less than 150% of baseline creatinine. AKI stage 1 was serum creatinine at presentation between 150% and 199% of baseline creatinine *and* at least 0.3 mg/dL higher than baseline. AKI stage 2 was serum creatinine at presentation between 200% and 299% of baseline *and* at least 0.3 mg/dL higher than baseline. AKI stage 3 was serum creatinine at presentation at least 300% of baseline.

We first tested for an interaction between the treatment and variable defining the subgroups in a logistic or linear mixed effects model with site included as a random effect. Analysis within subgroups were then performed for any variable showing an interaction yielding a p-value of 0.20 or less. Statistical methods for each outcome in the subgroup analysis were the same as used for the primary analysis.

O. Determination of Adverse Events

Adverse events (AEs) were queried from the medical record at the end of the intervention phase and between study days 5-7. We limited the time window for determination of AEs because we are unaware of any biologically plausible reason to anticipate that either fluid type should lead to AEs remote from the immediate intervention period. AEs that were unexpected, serious, and at least possibly related to study fluid type were reviewed by the international steering committee and reported promptly to regulatory bodies.

Figure S1. Study Overview

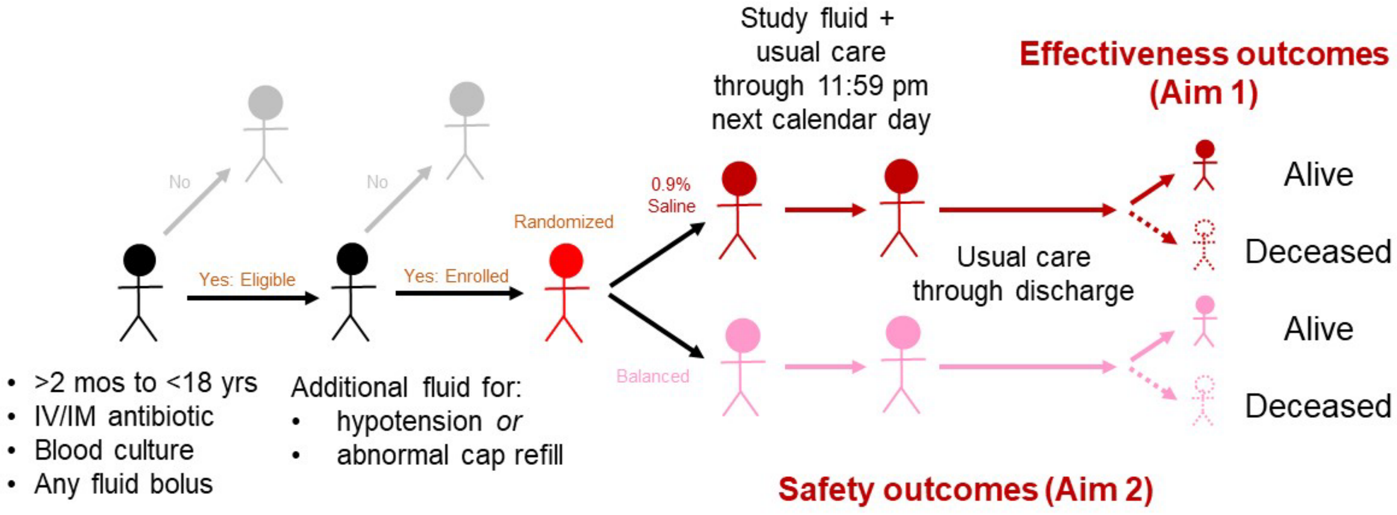

The figure illustrates the overall design and flow of participants through the study. Patients who were between 2 months and 18 years-old and treated for suspected septic shock, as indicated by clinical decision to collect a blood culture and administers a parenteral antibiotic dose, were eligible and enrolled if fluid bolus therapy was administered for abnormal perfusion (defined by either clinician judgement that hypotension or abnormal capillary refill (either "flash" or "prolonged") capillary refill were present). After randomization, patients were allocated to receive either balanced fluid or 0.9% saline for all subsequent fluid boluses and maintenance hydration through 11:59 pm on the following calendar day. All decisions about timing, volume, and rate of fluid administration and all other aspects of management remained at the discretion of the treating clinicians. Effectiveness and safety outcomes were assessed through hospital discharge or study day 30, whichever came first. Mortality was followed out to 90 days after randomization.

Figure S2. Projected and Actual Participant Enrollment

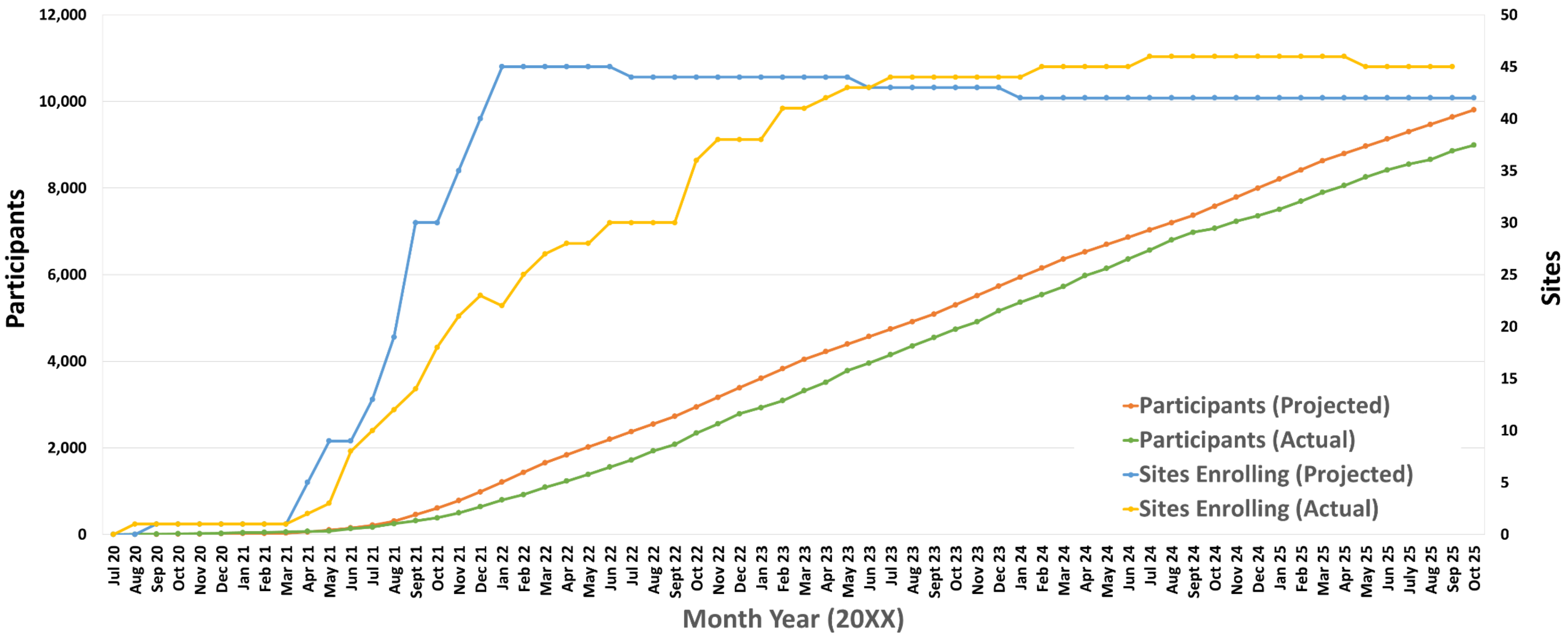

Enrollment began on August 25, 2020, and concluded on October 31, 2025. Initial enrollment was delayed at many sites due to the 2020 COVID pandemic. Enrollment was paused temporarily in the US in late 2024 through early 2025 after Hurricane Helene disrupted the availability of crystalloid fluids across sites. Although the total time for enrollment was extended by two months, the final enrollment of 9,041 patients was slightly less than the planned target of 9,179 patients.

Figure S3. Screening, Randomization, and Analysis

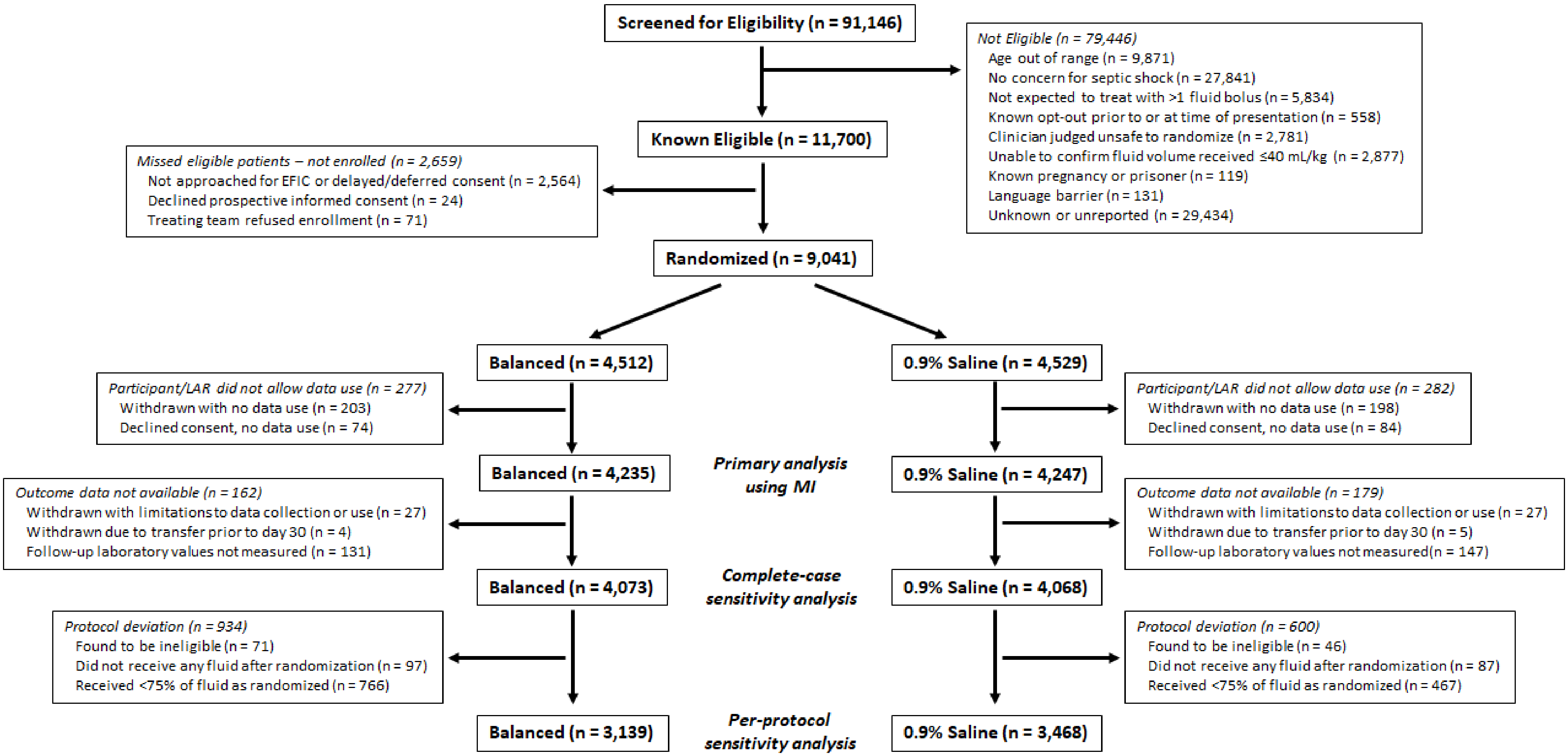

Patients presenting to a study site emergency department were screened for eligibility as part of routine clinical care. Screening results were recorded for patients identified as eligible for enrollment and a periodic sampling of patients not enrolled in the study. To identify ineligible and missed eligible patients, sites in the United States periodically reviewed their existing local sepsis-tracking systems, sites in Canada and Costa Rica reviewed

emergency department logs, and sites in Australia and New Zealand reviewed either their existing local sepsis-tracking systems, emergency department logs, or a computerized report to identify all emergency department patients who received intravenous antibiotics and any type of intravenous fluid bolus. Sites that enrolled fewer than 80% of eligible patients during any quarter were identified for additional education, training, and feedback to improve enrollment. Reasons that patients were not eligible or eligible but not enrolled are shown, with additional details provided in Table S3. Of the total 9,041 enrolled/randomized patients, 277 in the balanced fluid and 282 in the 0.9% saline groups withdrew from the study and did not allow use of their data. The primary analysis was performed using 4,235 patients assigned to balanced fluid and 4,247 assigned to 0.9% saline, including 162/4,235 and 179/4,247 patients, respectively, for whom some components of MAKE30 were missing. Multiple imputation (MI) was used to account for missing data with assumption that data were missing-at-random. Sensitivity analyses were performed using the subgroups of patients with complete MAKE30 outcome data and those who were adherent to the study protocol.

Figure S4. Type of Informed Consent

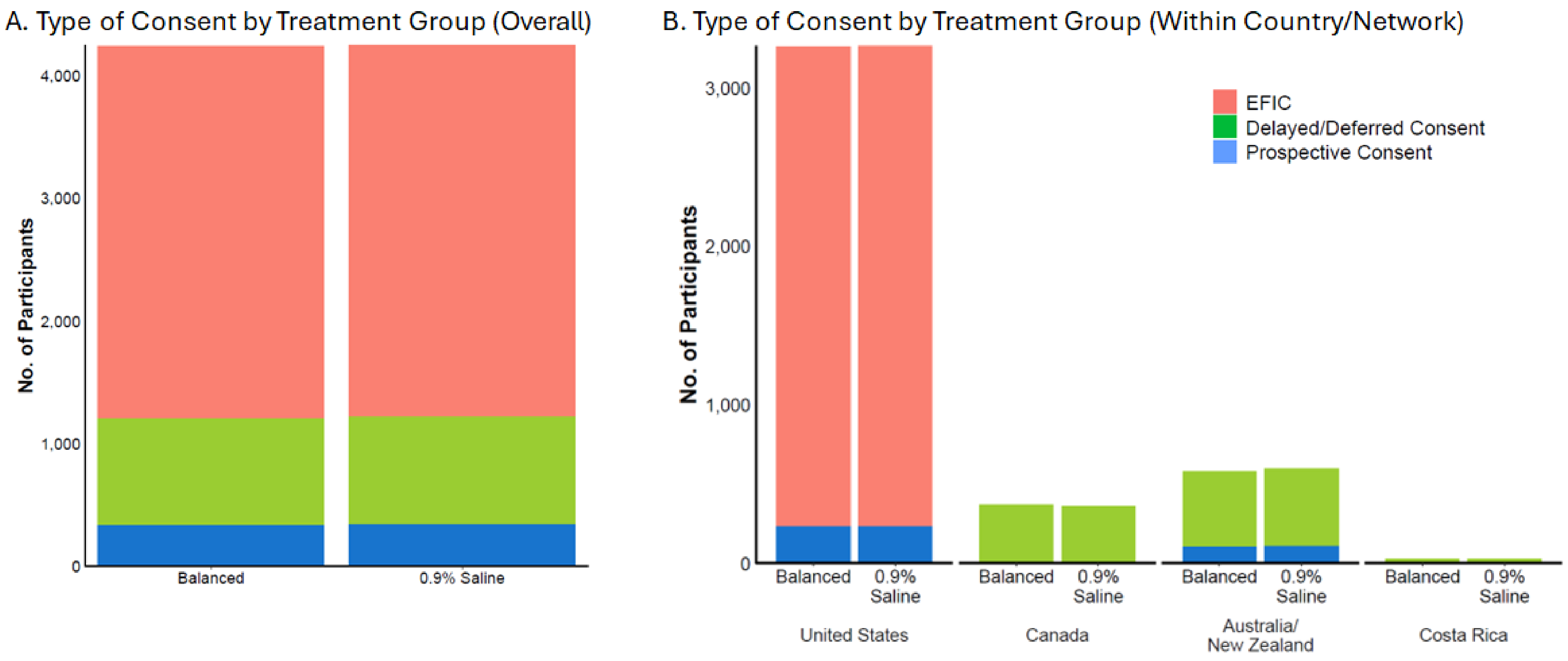

The number of participants who were enrolled in the study through prospective informed consent or the approved alternatives, including Exception from Informed Consent (EFIC) and delayed/deferred consent, are shown by treatment group both overall (A) and within each country/research network (B).

Figure S5. Fluid Volume by Study Phase

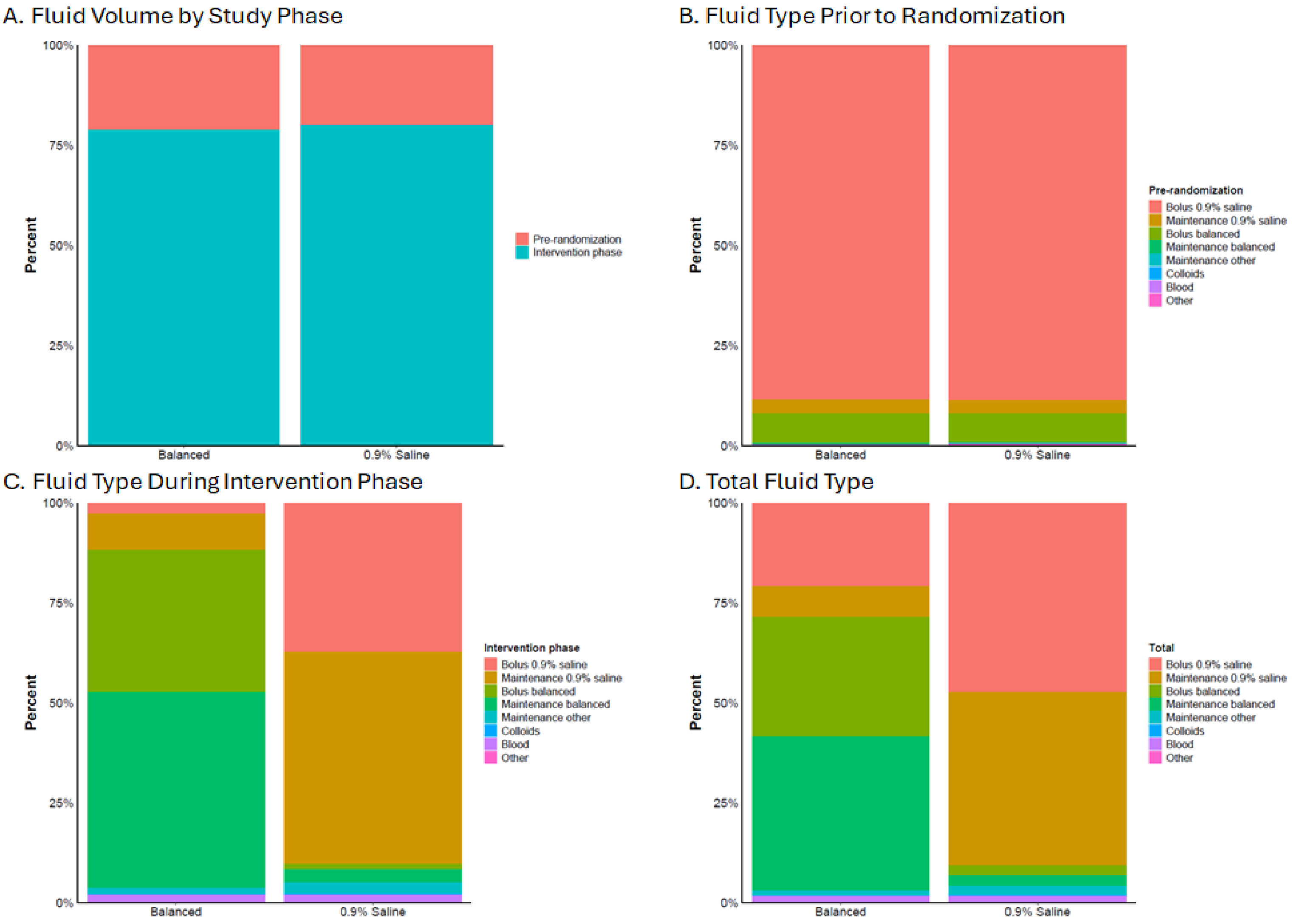

The mean percent of total fluid volume received by study patients, according to assigned treatment group, during the pre-randomization and intervention phases (A). The mean percent volume of each fluid type study patients received during the pre-randomization (B), intervention (C), and combined (D) phases, according to assigned treatment group.

Figure S6. Components of the MAKE30 Composite Outcome

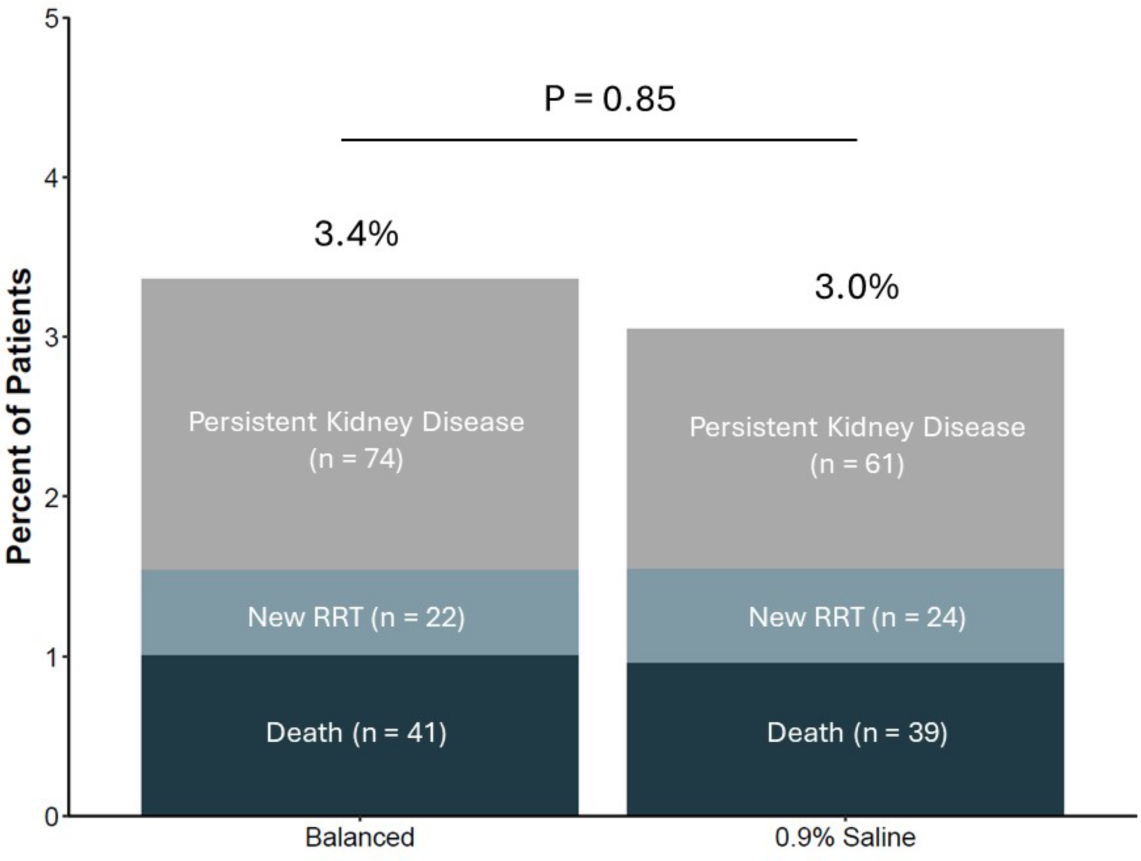

For the balanced fluid (left) and 0.9% saline (right) groups, the number of patients (with percentages on the y-axis) who experienced death from any cause, new renal replacement therapy (RRT, among survivors), and persistent renal dysfunction (among survivors without new RRT) within 30 days of study enrollment or hospital discharge, whichever came first, are shown. The overall incidence of Major Adverse Kidney Events within 30 days (MAKE30) was 3.4% in the balanced fluid group and 3.0 % in the saline group. The P value is for the primary analysis after multiple imputation for missing data using the Cochran–Mantel–Haenszel test, stratified by study site.

Figure S7: Results of the Tipping Point Analysis for MAKE30

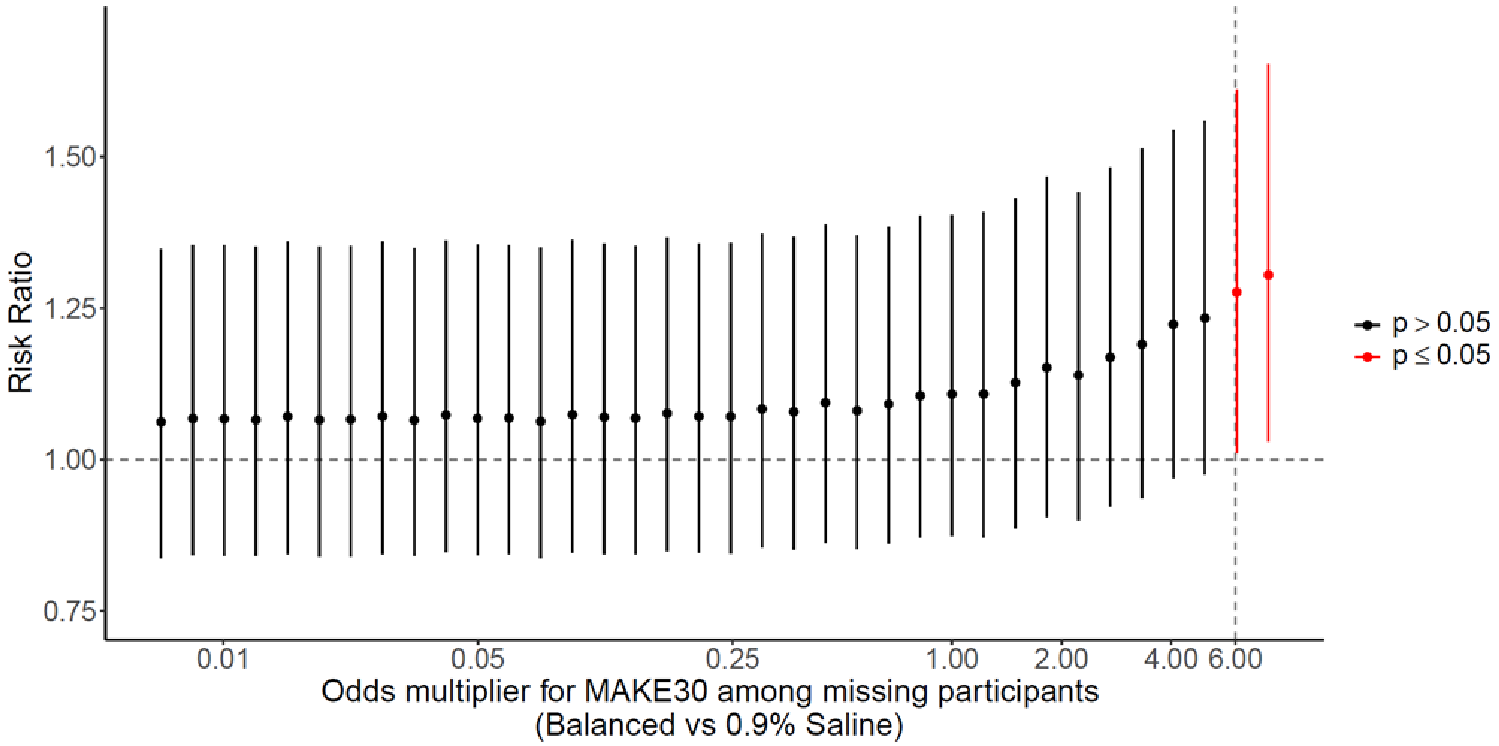

Across the prespecified grid of δ values, the pooled treatment effect for MAKE30 remained statistically non-significant over a wide range of departures from the missing-at-random (MAR) assumption. The 95% confidence interval did not exclude the null until δ approached approximately 1.8–2.0 on the log-odds scale, corresponding to an approximately 6-fold increase in the odds of MAKE30 among participants with originally missing outcomes in the balanced fluid group (i.e., $e^{\delta} \geq 6$). Thus, the primary findings would change only under an extreme and clinically implausible scenario in which participants with missing outcomes in the balanced group had substantially higher MAKE30 risk than predicted under MAR. Overall, the tipping-point analysis supports the robustness of the primary results to reasonable departures from the MAR assumption.

Figure S8. Mortality Within 90 Days

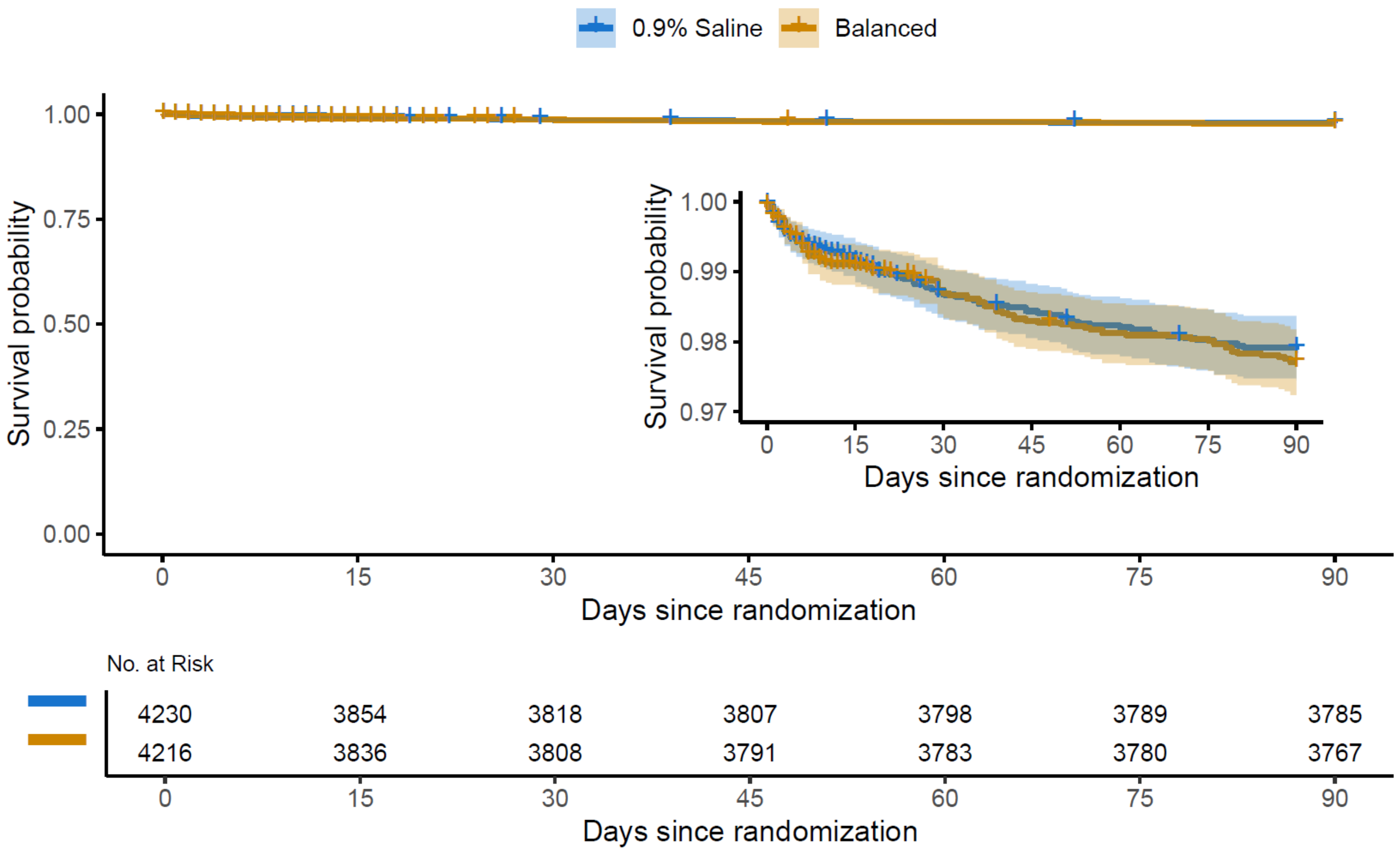

Kaplan‑Meier estimates of the probability of survival through 90 days following study enrollment were similar among the patients in the balanced fluid and 0.9% saline groups (hazard ratio for death from any cause, 1.10; 95% CI, 0.81 to 1.58; P=0.52). The proportional-hazards assumption, assessed by visual inspection of log–log survival plots and by testing Schoenfeld residuals (p=0.46), was not violated. The inset figure provides a magnification of the same results.

Figure S9. Box Plot of Laboratory Values by Treatment Arm

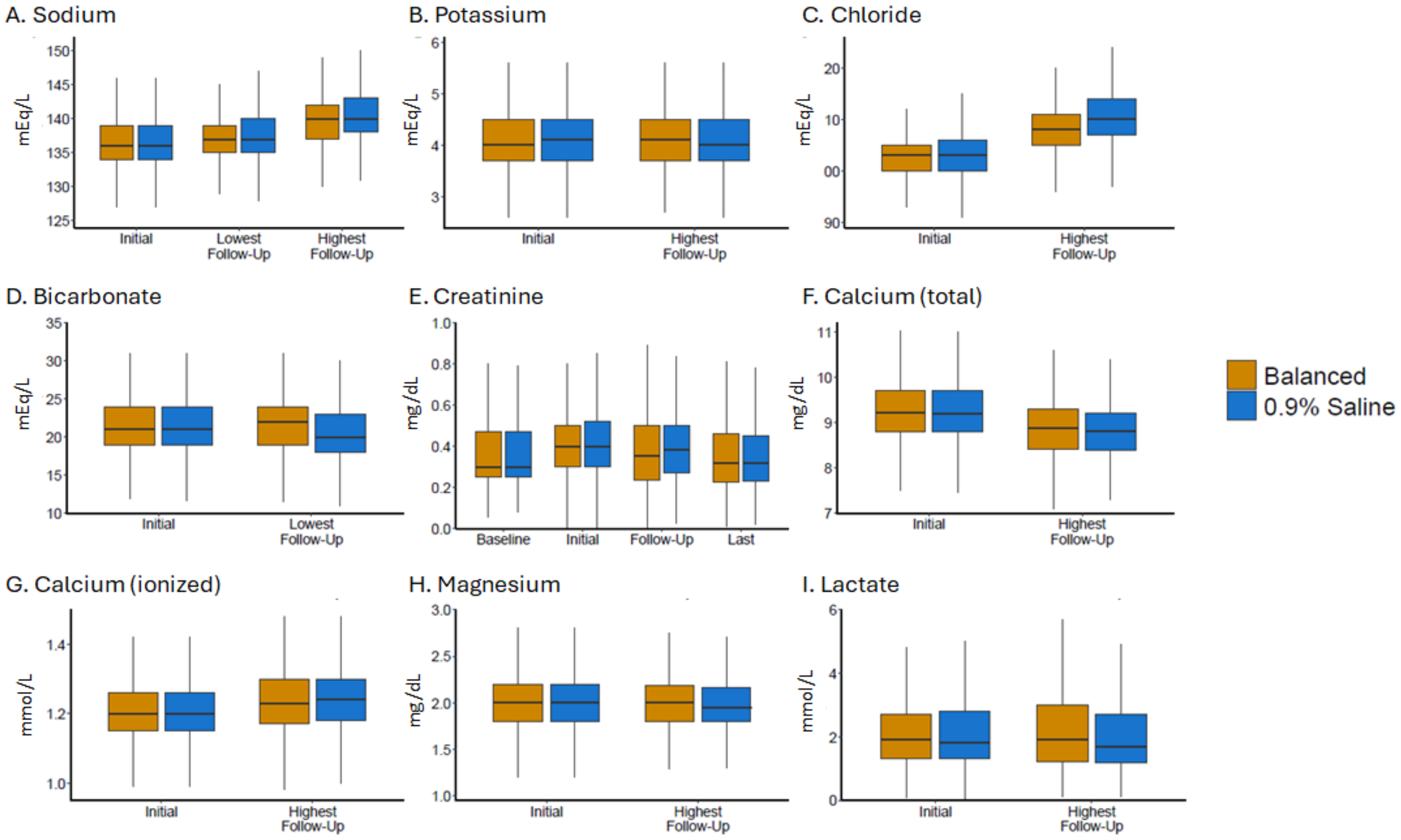

Shown are the median values for blood sodium, potassium, chloride, bicarbonate, creatinine, total calcium, ionized calcium, magnesium, and lactate at initial presentation and the highest follow-up value available from clinician-ordered measurements between 2 hours after randomization through study day 3. For sodium, the lowest follow-up value available from between 2 hours after randomization through study day 3 is also presented. For creatinine, the baseline (measured or imputed) and last measured creatinine values prior to hospital discharge or study day 30 are also presented. Data are presented as boxplots with median, interquartile range, and whiskers indicating values +/- 1.5 times the 25% and 75% percentiles (outliers beyond these thresholds are not shown).

Figure S10. Results of the Tipping Point Analysis for Safety Outcomes

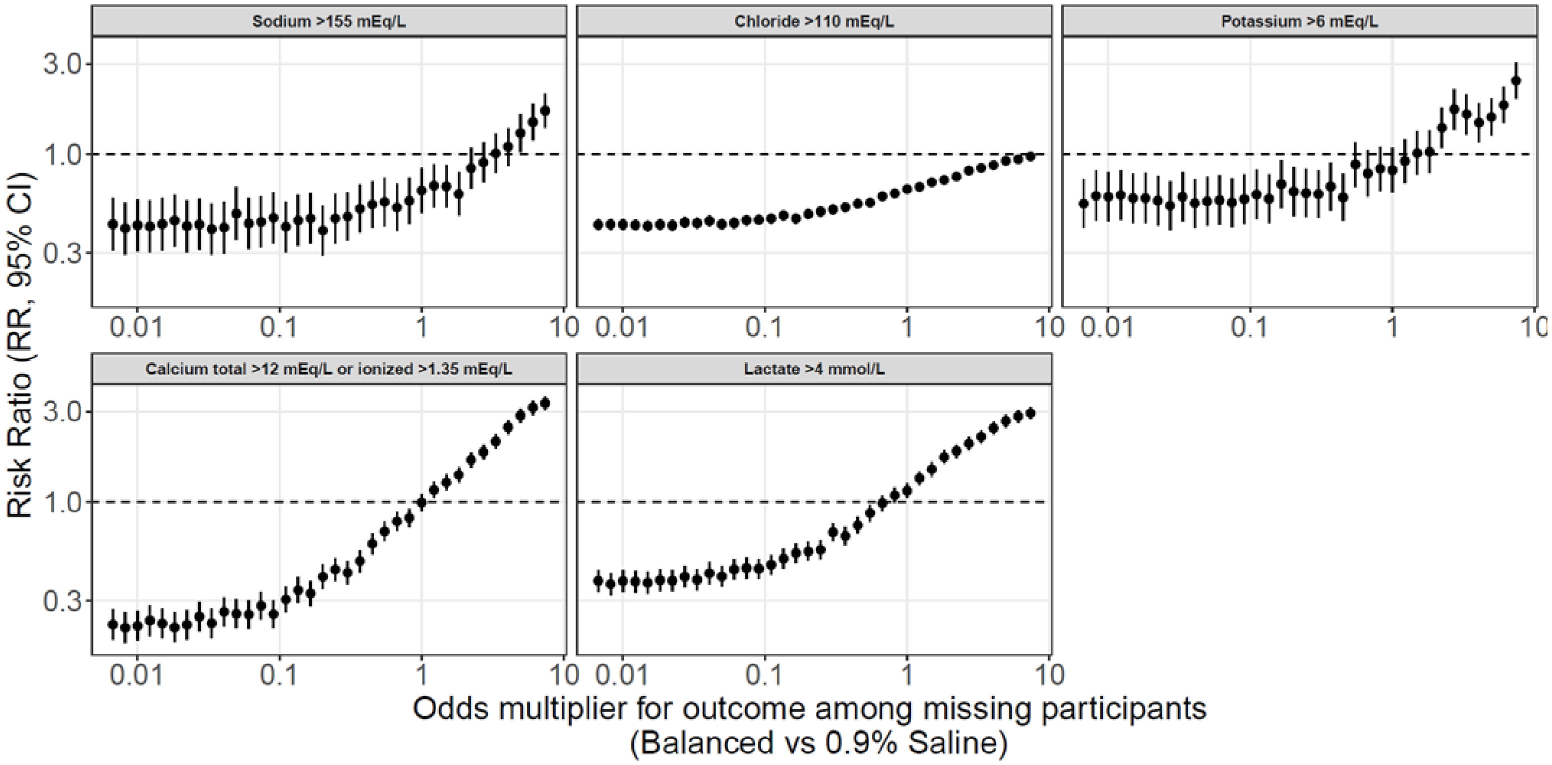

A tipping-point analysis was conducted for safety outcomes with more than 30% missing data. Missing data at this level only occurred in laboratory-based outcomes due to the clinical judgement of the treating clinician. Results are not shown for the safety outcome of "sodium <128 mEq/L" due to sparse events and resulting instability with extremely wide confidence intervals.

Figure S11. Chloride Concentration Relative to Volume of Crystalloid

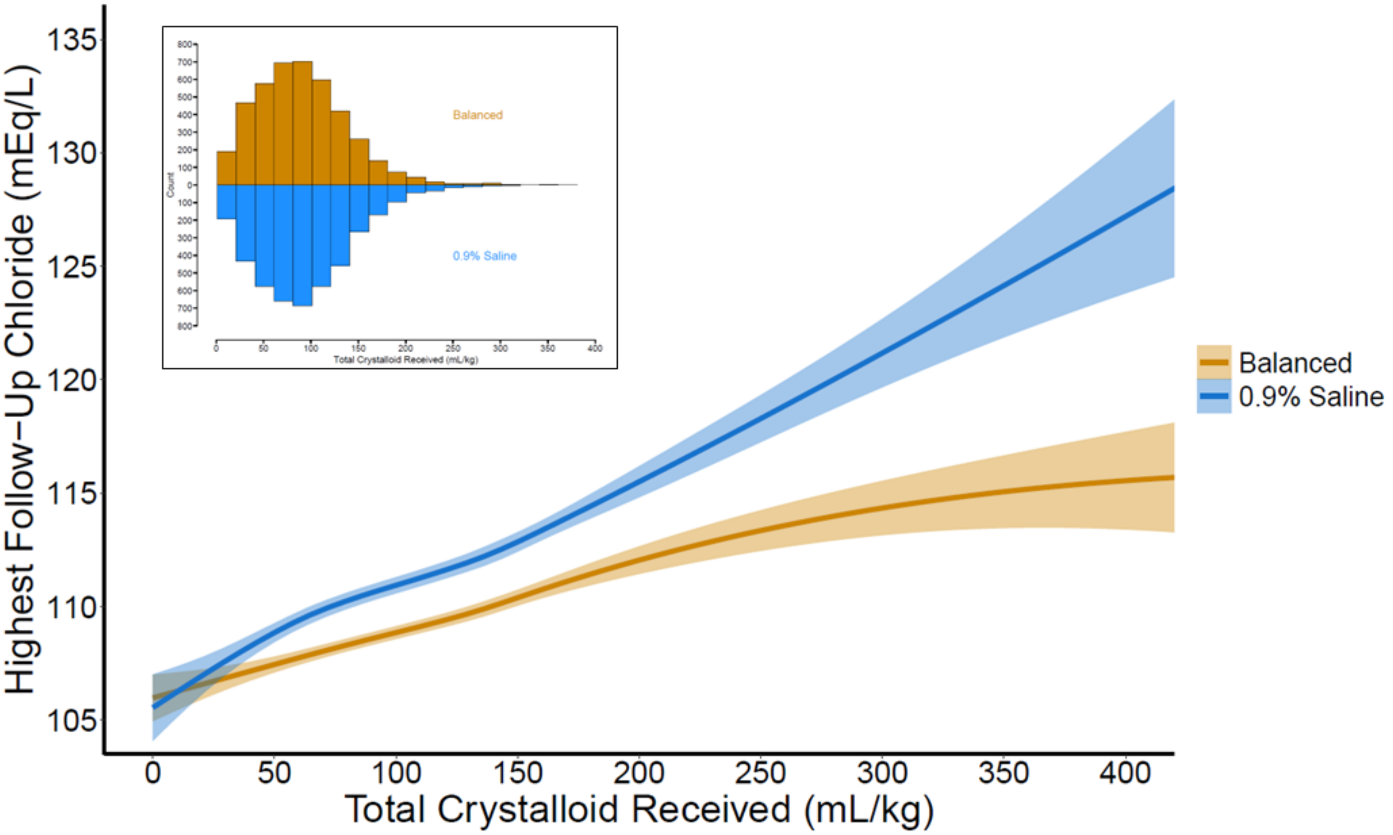

The highest blood chloride concentration between 2 hours after randomization through study day 3 was compared between patients assigned to balanced fluid (orange) and 0.9% saline (blue) groups relative to the total volume of crystalloid received (in mL/kg) during the pre-randomization and intervention phases combined. We used generalized additive models with penalized cubic regression splines to evaluate the non-linear association, allowing separate smooth functions by treatment group. Smoothed curves with 95% confidence intervals are displayed graphically. The inset mirror histogram shows the number of patients in each group who received each volume of total crystalloid fluid (using bins of successive 20 mL/kg volume increments). Data for patients who received >400 mL/kg of total crystalloid fluid volume were excluded due to low frequencies.

Figure S12. Total CO2 (Bicarbonate) Concentration Relative to Volume of Crystalloid

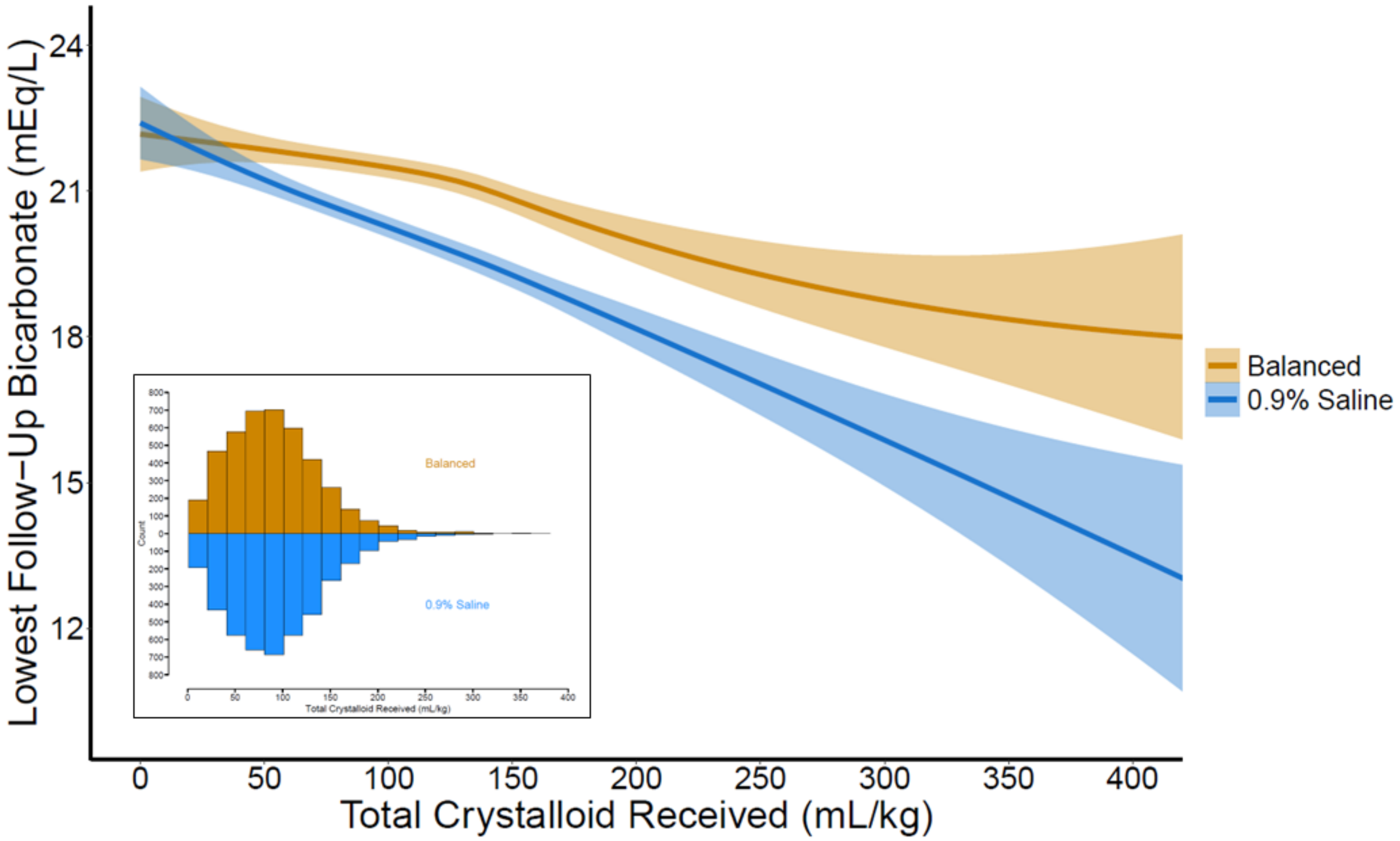

The lowest blood bicarbonate concentration between 2 hours after randomization through study day 3 was compared between patients assigned to balanced fluid (orange) and 0.9% saline (blue) groups relative to the total volume of crystalloid received (in mL/kg) during the pre-randomization and intervention phases combined. We used generalized additive models with penalized cubic regression splines to evaluate the non-linear association, allowing separate smooth functions by treatment group. Smoothed curves with 95% confidence intervals are displayed graphically. The inset mirror histogram shows the number of patients in each group who received each volume of total crystalloid fluid (using bins of successive 20 mL/kg volume increments). Data for patients who received >400 mL/kg of total crystalloid fluid volume were excluded due to low frequencies.

Figure S13. Serum Creatinine Relative to Volume of Crystalloid

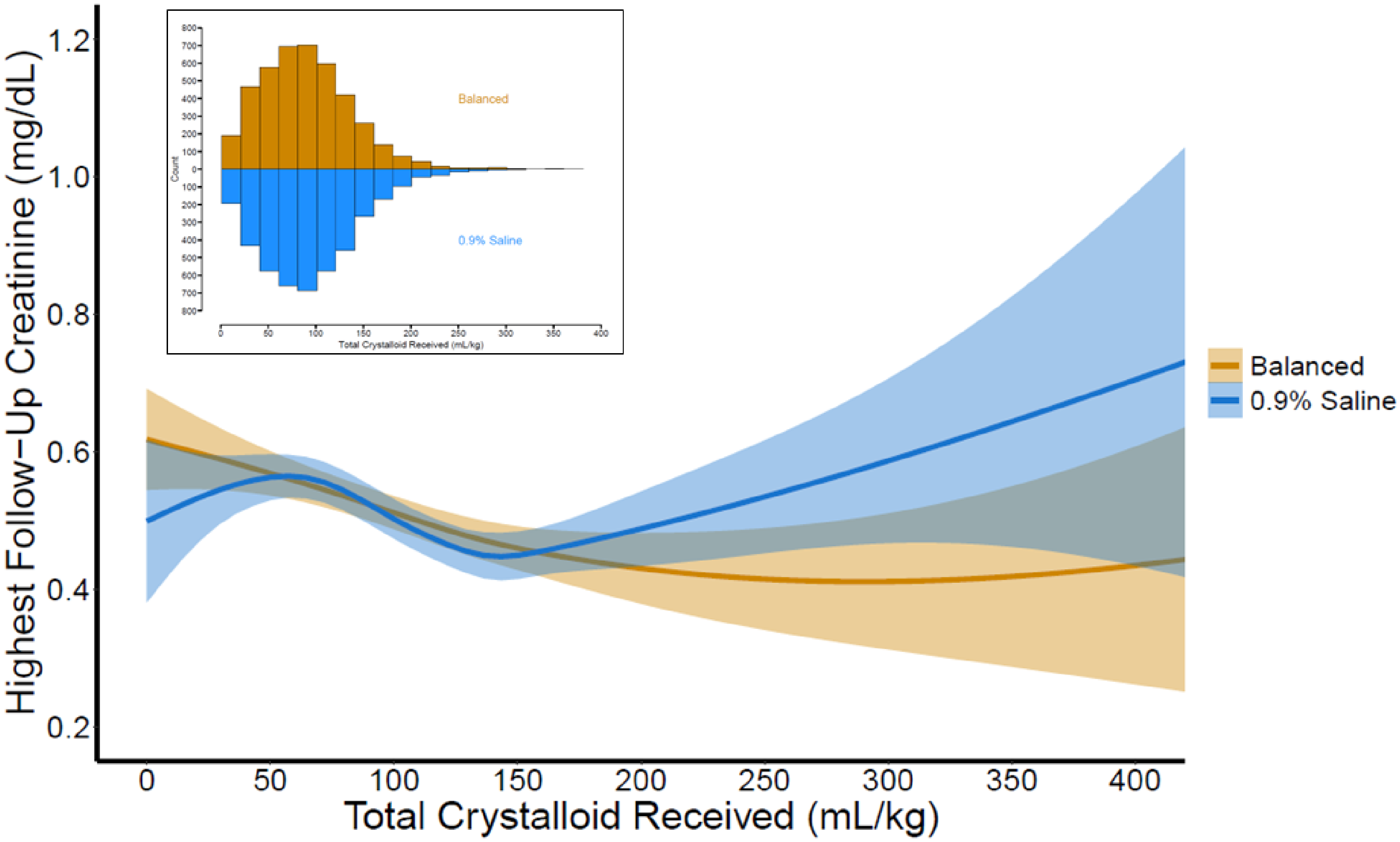

The highest serum creatinine measured between 2 hours after randomization through study day 3 was compared between patients assigned to balanced fluid (orange) and 0.9% saline (blue) groups relative to the total volume of crystalloid received (in mL/kg) during the pre-randomization and intervention phases combined. We used generalized additive models with penalized cubic regression splines to evaluate the non-linear association, allowing separate smooth functions by treatment group. Smoothed curves with 95% confidence intervals are displayed graphically. The inset mirror histogram shows the number of patients in each group who received each volume of total crystalloid fluid (using bins of successive 20 mL/kg volume increments). Data for patients who received >400 mL/kg of total crystalloid fluid volume were excluded due to low frequencies

Figure S14: Subgroup Analysis of MAKE30 by Measured versus Imputed Baseline Serum Creatinine

	Balanced Fluid	0.9% Saline	Risk Ratio (95% CI)
	no. of events/total no. (%)		
**Baseline creatinine**			
Measured	101/2448 (4.1)	88/2462 (3.6)	1.21 (0.93−1.57)
Imputed	36/1625 (2.2)	36/1606 (2.2)	0.95 (0.63−1.43)
**Overall**	137/4073 (3.4)	124/4068 (3.0)	1.10 (0.88−1.40)

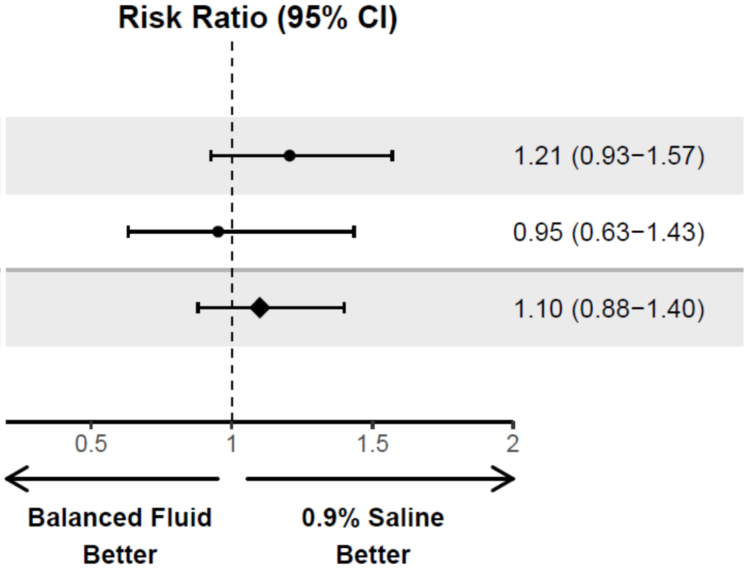

The risk ratio and 95% confidence interval are shown according to subgroups who had a measured or imputed values for baseline serum creatinine. Baseline serum creatinine was recorded as the lowest value available between 12 months and 24 hours prior to study enrollment. If no such value was available in this time window, baseline serum creatinine was imputed using median creatinine values for age and sex as described in the Supplementary Appendix.

Figure S15: Additional Subgroup Analyses of MAK30 by Initial Illness Severity

	Balanced Fluid	0.9% Saline	Risk Ratio (95% CI)
	no. of events/total no. (%)		
**Initial bicarbonate**			
>=20 mEq/L	74/2565 (2.9)	63/2533 (2.5)	1.16 (0.85−1.60)
15−19 mEq/L	34/939 (3.6)	29/970 (3.0)	1.28 (0.78−2.09)
<15 mEq/L	19/195 (9.7)	26/205 (12.7)	0.92 (0.53−1.60)
Not measured	10/374 (2.7)	6/360 (1.7)	1.02 (0.59−1.75)
**Initial blood lactate**			
<2 mmol/L	29/1269 (2.3)	25/1292 (1.9)	1.21 (0.75−1.94)
2−5 mmol/L	40/1068 (3.7)	37/1022 (3.6)	1.04 (0.68−1.61)
>5 mmol/L	21/198 (10.6)	27/203 (13.3)	0.76 (0.45−1.29)
Not measured	47/1538 (3.1)	35/1551 (2.3)	1.23 (0.86−1.76)
**Overall**	137/4073 (3.4)	124/4068 (3.0)	1.10 (0.88−1.40)

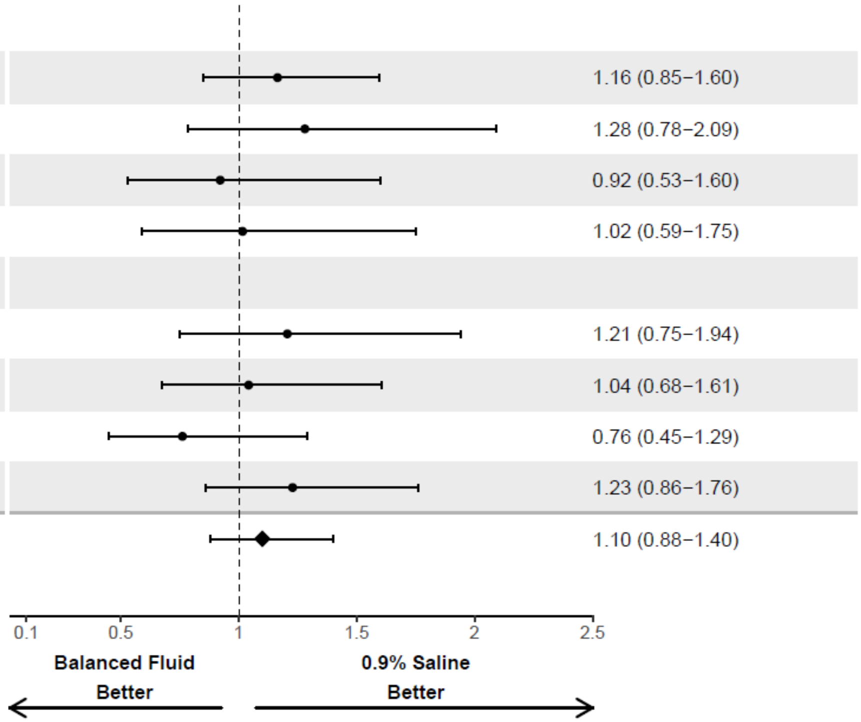

The risk ratio and 95% confidence interval are shown according to subgroup for the percentage of patients in the balanced fluid and 0.9% saline groups who met criteria for the composite outcome of a major adverse kidney event within 30 days (defined as death from any cause, new renal replacement therapy, or persistent kidney dysfunction). Initial bicarbonate concentration and initial blood lactate were values measured closet to randomization. “Not measured” included patients for whom the laboratory value was not measured and was evaluated separately.

Figure S16. Heterogeneity of Treatment Effect of MAKE30 by Fluid Volume

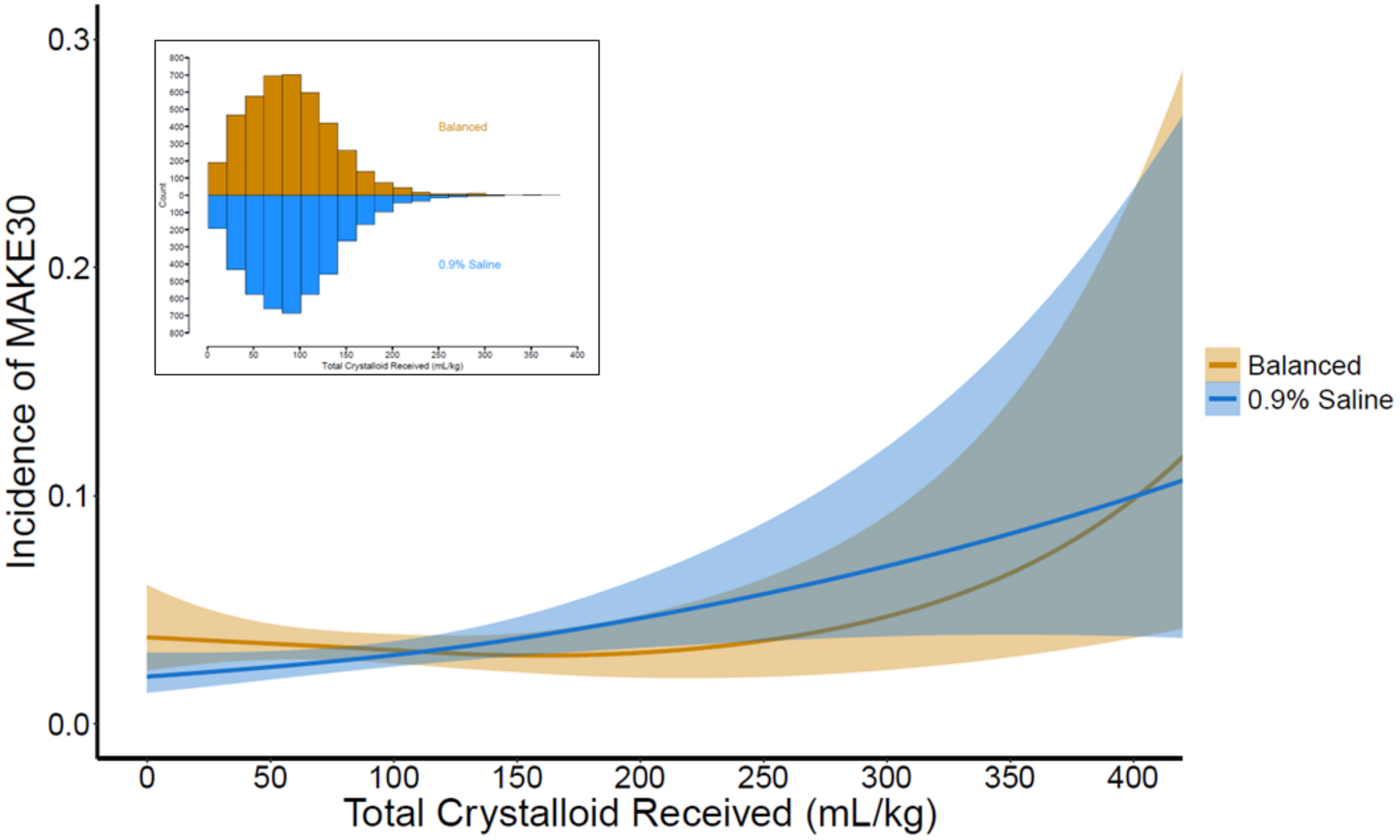

The figure displays the primary outcome of Major Adverse Kidney Events within 30 days (MAKE30) for patients assigned to the balanced fluid and 0.9% saline groups relative to the total volume of crystalloid received (in mL/kg) during the pre-randomization and intervention phases combined. Smoothed curves with 95% confidence intervals are displayed graphically. The inset mirror histogram shows the number of patients in each group who received each volume of total crystalloid fluid (using bins of successive 20 mL/kg volume increments). Data for patients who received >400 mL/kg of total crystalloid fluid volume were excluded due to low frequencies.

Table S1. Composition of Human Plasma and Crystalloid Fluids

	Sodium	Potassium	Chloride	Calcium	Magnesium	Lactate	Acetate	Gluconate	Osmolality
Human plasma	135-145	3.5-5.0	94-110	4.3-5.3	1.5-2.5	0-2			275-295
0.9% saline	154		154						308
Balanced fluids									
Lactated Ringer's	130	4.0	109	2.7		28			273
Plasma-Lyte	140	5.0	98		3.0		27	23	294
Hartmann's solution	131	5.0	111	2.7		29			278

All values are in mEq/L except osmolarity, which is in mOsm/L. Actual electrolyte composition may vary slightly in some fluid types depending on country and manufacturer.

Table S2. Study Procedures and Timeline

<table>
<tr><th></th><th>Screening</th><th colspan="2">Intervention Phase</th><th>Follow-Up[a]</th></tr>
<tr><td>Study Day</td><td>0</td><td>0</td><td>1</td><td>2 to 90</td></tr>
<tr><td>ENROLLMENT:</td><td></td><td></td><td></td><td></td></tr>
<tr><td>Eligibility screen</td><td>X</td><td></td><td></td><td></td></tr>
<tr><td>Informed consent or enrolment under EFIC or deferred/delayed consent to continue</td><td>X</td><td></td><td></td><td></td></tr>
<tr><td>Treatment allocation</td><td></td><td>X</td><td></td><td></td></tr>
<tr><td>Inform patient/LAR of study enrollment if EFIC or deferred/delayed consent</td><td></td><td colspan="2">X[b]</td><td></td></tr>
<tr><td>Biological sample informed consent</td><td></td><td colspan="3">X[c]</td></tr>
<tr><td>INTERVENTIONS:</td><td></td><td></td><td></td><td></td></tr>
<tr><td>Balanced crystalloid fluid (intervention)</td><td></td><td colspan="2">———</td><td></td></tr>
<tr><td>0.9% saline (control)</td><td></td><td colspan="2">———</td><td></td></tr>
<tr><td>ASSESSMENTS:</td><td></td><td></td><td></td><td></td></tr>
<tr><td>Baseline demographics</td><td></td><td>X</td><td></td><td></td></tr>
<tr><td>Medical history</td><td></td><td>X</td><td></td><td>X</td></tr>
<tr><td>Fluid administration</td><td></td><td>X</td><td>X</td><td></td></tr>
</table>

Concurrent therapies		X	X		
Laboratory data		X	X		
Urine and blood collection		X[d,g]		X[e,g]	X[f,g]
Effectiveness endpoints				X	
Safety endpoints			X	X	
Adverse events			X	X	

[a] Follow-up stopped at 90 days after study enrollment

[b] Post-enrollment discussion with LAR occurred as soon as feasible after study enrollment

[c] For study participants enrolled at three sites in the United States, separate informed consent for collection of blood and urine was obtained as early as feasible after study enrolment

[d] Urine and blood collection on day 0 (i.e., day of randomization) preferred but up to day 1 allowable (as close to the time of randomization as feasible)

[e] Urine and blood collection on day 2 (i.e., the first calendar day of the follow-up phase), up to day 3 allowed

[f] Urine and blood collection on day 27, or prior to expected discharge or death, whichever comes first)

[g] If the study team is unable to obtain the research samples (blood and urine), leftover or remnant specimen (blood and urine) will be collected from the clinical lab to use for the research.

Table S3. Detailed Reasons for Not Eligible and Missed Eligible Patients

Reason	Frequency
Not Eligible	79,446
Age out of range (n = 9,871)	9,871/79,446
No concern for septic shock (n = 27,841)	27,841/79,446
Not expected to treat with >1 fluid bolus (n = 5,834)	5,834/79,446
Known opt-out prior to or at time of presentation (n = 558)	558/79,446
Clinician judged unsafe to randomize (n = 2,781)	2,781/79,446
Suspicion for impending brain herniation	*56/2,781*
Known hyperkalemia (serum or whole blood potassium >6 mEq/L)	*163/2,781*
Known hypercalcemia (total calcium >12 mg/dL or ionized calcium > 1.35 mmol/L)	*66/2,781*
Known acute fulminant hepatic failure (alanine aminotransferase >10,000 U/L or total bilirubin >12.0 mg/dL)	*90/2,781*
Known history of severe hepatic impairment, defined as diagnosis of cirrhosis or liver failure, or active listing for liver transplant	*37/2,781*
Known history of severe kidney disease, defined as current dependency on peritoneal dialysis or hemodialysis	*513/2,781*
Other concern about chronic kidney impairment (e.g., prior kidney transplant)	*336/2,781*
Known metabolic disorder, inborn error of metabolism, or primary mineralocorticoid deficiency (e.g., mitochondrial disorder, urea cycle disorder, amino acidemia, fatty acid oxidation disorder, glycogen storage disorder, congenital adrenal hypoplasia, Addison's disease)	*958/2,781*
Known new oncologic diagnosis	*2/2,781*

Known use of ketogenic diet	*9/2,781*
Unknown clinical safety concern	*551/2,781*
Unable to confirm fluid volume received ≤40 mL/kg (n = 2,877)	2,877/79,446
Known pregnancy or prisoner (n = 119)	119/79,446
Language barrier (n = 131)	131/79,446
Unknown or unreported (n = 29,434)	29,434/79,446
Missed Eligible	2,659
Not approached for EFIC or delayed/deferred consent process	2,564/2,659
Treating clinician not approved to enroll/not on study team	*92/2,564*
Insufficient time to enroll	*92/2,564*
Unknown reason not approached	*2,380/2,564*
Declined prospective informed consent	24/2,659
Treating team refused enrollment	71/2,659

Table S4. Country of Enrollment

Country	Balanced Fluid	0.9% Saline
	(n =4,235)	(n = 4,247)
United States – n (%)	3,262 (77)	3,264 (77)
Canada – n (%)	367 (8.7)	360 (8.5)
Australia or New Zealand – n (%)	579 (14)	595 (14)
Costa Rica – n (%)	27 (0.64)	28 (0.66)

Table S5. Additional Demographic Characteristics of Participants

Characteristic	Balanced Fluid	0.9% Saline
	(n =4,235)	(n = 4,247)
Age category – no. (%)		
2 to <6 months	128 (3.0)	124 (2.9)
6 months to <1 year	223 (5.3)	235 (5.5)
1 to <5 years	1,328 (31)	1,342 (32)
5 to <12 years	1,360 (32)	1,330 (31)
12 to <18 years	1,176 (28)	1,202 (28)
≥18 years	17 (<1)	9 (<1)
Missing	3 (<1)	5 (<1)
Race – no. (%)		
White	1,925 (45)	1,825 (43)
Black	484 (11)	510 (12)
Asian	146 (3.4)	168 (4.0)
American Indian/Alaska Native	23 (0.54)	26 (0.61)
Native Hawaiian/Other Pacific Islander	17 (0.40)	22 (0.52)
Multiple races reported	67 (1.6)	57 (1.3)
Unknown/Not reported[a]	1,573 (37)	1,639 (39)
Ethnicity		
Not Hispanic or Latino – n (%)[b]	2,154 (51)	2,161 (51)
Hispanic or Latino – n (%)[b]	1,030 (24)	1,013 (24)
Aboriginal or Torres Strait Islander – n (%)[c]	27 (0.64)	34 (0.80)

New Zealand Māori – n (%)[c]	19 (0.45)	34 (0.80)
Other – n (%)[d]	534 (13)	526 (12)
Unknown/Not reported – n (%)[e]	471 (11)	479 (11)

[a] Race was not collected from participants enrolled at sites in Canada, Australia, or New Zealand

[b] Hispanic or Latino ethnicity was reported in the United States, only

[c] Aboriginal or Torres Strait Islander or New Zealand Māori was reported in Australia/New Zealand, only

[d] "Other" ethnicity included self-report from patients enrolled in the United States or Australia/New Zealand

[e] Ethnicity was not able to be collected from participants enrolled in Canadian sites

Table S6. Site of Infection

Site of Infection – no. (%)	Balanced Fluid	0.9% Saline
Primary bloodstream	364 (8.6)	344 (8.1)
Pneumonia or other lung infection	2,002 (47)	1,948 (46)
Abdominal	349 (8.3)	406 (9.6)
Genitourinary	361 (8.5)	375 (8.9)
Central nervous system	57 (1.3)	60 (1.4)
Skin/soft tissue	180 (4.3)	182 (4.3)
Other infection	99 (2.3)	104 (2.5)
Unknown site	565 (13)	575 (14)
Alternative diagnosis (not infection/sepsis)	247 (5.8)	242 (5.7)
Missing	11	11
Any positive blood culture, primary or secondary – no. (%)	519 (12)	478 (11)
Missing	11	13

Table S7. Alternative Diagnoses for Participants Found Not to Have Sepsis

Condition	Frequency
Multisystem inflammatory syndrome in children (MIS-C)[a]	67
Acute or progressive neurologic condition	56
Hypovolemia/hypovolemic shock	38
Gastrointestinal, medical disease	36
Febrile neutropenia	22
Kawasaki disease	21
Rheumatologic condition, new or flare	20
Appendicitis	16
Gastrointestinal, surgical disease	16
Acute respiratory failure	16
Ingestion/intoxication	16
Fever, not otherwise specified	15
New-onset oncologic condition	14
Anemia	10
Dysautonomia/Paroxysmal sympathetic hyperactivity	8
Hypoglycemia	8
Heart failure/cardiogenic shock	8
Sickle cell complication	7
Cerebrospinal fluid shunt malfunction	6
Local infection	6
Anaphylaxis	5

Botulism	5
Acute on chronic respiratory failure	5
Aspiration	5
Adrenal insufficiency	5
Drug fever	4
Hypothermia, not otherwise specified	4
Pericarditis/pericardial effusion	4
Pancreatitis	4
Post-operative fever	4
Diabetic ketoacidosis	4
Other/unknown	4
Inflammatory disorder of the skin	3
Cytokine release syndrome	3
Hemophagocytic lymphohistiocytosis	2
Shock, not otherwise specified	2
Acute on chronic kidney failure	2
Foreign body	2
Splenic laceration	2
Envenomation	2
Gonadal torsion	2
Pulmonary hypertension	1
Pulmonary alveolar proteinosis	1
Acute liver failure	1

Lyme disease	1
Diffuse alveolar hemorrhage	1
Heat stroke	1
Hemorrhage, not otherwise specified	1
Pulmonary embolism	1
Ureteropelvic junction obstruction	1
Hypocalcemia	1
Retained products of conception	1
Post-cardiac arrest syndrome	1
Hemorrhagic edema of infancy	1
Dengue fever	1

[a] Multisystem inflammatory syndrome in children (MIS-C) includes Pediatric Inflammatory Multisystem Syndrome Temporally associated with SARS-CoV-2 (PIMS-TS) as alternative name for this condition.

Table S8. KDIGO AKI Stage by Measured or Imputed Baseline Serum Creatinine

KDIGO AKI Stage at Study Enrollment	Balanced Fluid	0.9% Saline
	(n =4,235)	(n = 4,247)
Measured baseline creatinine – no. (%)	2,515 (59)	2,529 (60)
None	1,782 (71)	1,752 (69)
Stage 1	455 (18)	461 (18)
Stage 2	137 (5.4)	154 (6.1)
Stage 3	141 (5.6)	162 (6.4)
Imputed baseline creatinine – no. (%)	1,717 (41)	1,714 (40)
None	1,485 (86)	1,437 (84)
Stage 1	105 (6.1)	151 (8.8)
Stage 2	64 (3.7)	60 (3.5)
Stage 3	63 (3.7)	66 (3.9)

AKI, acute kidney injury; KDIGO, Kidney Disease: Improving Global Outcomes

Table S9. Treatments for Sepsis from Presentation Through End of Intervention Phase

Therapy	Balanced Fluid	0.9% Saline
	(n =4,235)	(n = 4,247)
Ceftriaxone – n (%)[a]	2,593 (61)	2,654 (63)
Vasoactive medication(s) – n (%)[b,c]	599 (14)	608 (14)
Corticosteroids – n (%)[b]	956 (23)	923 (22)
Bicarbonate or other buffer – n (%)[b]	194 (4.6)	249 (5.9)
Invasive mechanical ventilation – n (%)[b,d]	410 (9.7)	412 (9.7)
Extracorporeal membrane oxygenation – n (%)[b]	26 (<1)	20 (<1)

[a] Data were missing from 7 participants in the 0.9% saline group and 7 participants in the balanced fluid group

[b] Data were missing from 11 participants in the 0.9% saline group and 11 participants in the balanced fluid group

[c] Vasoactive medications include continuous infusions of epinephrine, norepinephrine, dopamine, dobutamine, vasopressin, milrinone, and/or phenylephrine

[d] Includes only invasive mechanical ventilation delivered through an endotracheal tube (oral or nasal), tracheostomy, laryngeal mask airway, or other invasive device

Table S10. Fluid Administration

Characteristic	Balanced Fluid	0.9% Saline	Absolute Difference (95% CI)[a]
Number of crystalloid fluid boluses – median (IQR)			
Total[b]	2.0 [2.0, 3.0]	2.0 [2.0, 3.0]	0 (0 to 0)
Prior to randomization	1.0 [0, 1.0]	1.0 [0, 1.0]	0 (0 to 0)
During intervention phase	1.0 [1.0, 2.0]	1.0 [1.0, 2.0]	0 (0 to 0)
Crystalloid volume administered (mL/kg) – median (IQR)[c]			
Total fluid (bolus and maintenance)[b]			
Any crystalloid	85 [55, 119]	88 [57, 123]	-2.3 (-4.3 to -0.30)
0.9% saline	20 [5.5, 32]	79 [49, 113]	-57 (-59 to -56)
Balanced fluid	58 [31, 92]	0 [0, 0]	54 (52 to 55)
Bolus fluid only			
Prior to randomization	19 [0, 20]	19 [0, 20]	0 (0 to 0)
During intervention phase	20 [11, 40]	20 [12, 40]	0 (0 to 0)
0.9% saline	19 [0, 20]	40 [20, 50]	-20 (-20 to -20)
Balanced fluid	20 [10, 40]	0 [0, 0]	20 (20 to 20)
Maintenance fluid only			

Any crystalloid	45 [22, 71]	47 [24, 75]	-1.3 (-3.0 to 0)
0.9% saline	0 [0, 2.7]	41 [16, 68]	-35 (-36 to -33)
Balanced fluid	35 [7.4, 61]	0 [0, 0]	32 (31 to 33)
Other	0 [0, 0]	0 [0, 0]	0 (0 to 0)
Proportion of total balanced fluid received as:			
Lactated Ringer's or Hartmann's solution – mean ± SD	0.81 ± 0.39	0.83 ± 0.37	0 (0 to 0)
Plasma-Lyte – mean ± SD	0.17 ± 0.37	0.17 ± 0.37	0 (0 to 0)
Proportion who received any colloid volume – no. (%)	62 (1.5)	65 (1.5)	-0.001 (-0.006 to 0.005)
Colloid volume administered (mL/kg) – median (IQR)	0 [0, 0]	0 [0, 0]	0 (0 to 0)
Proportion who received any blood product volume – no. (%)	394 (9.3)	419 (9.9)	-0.006 (-0.018 to 0.007)
Blood product volume administered (mL/kg) – median (IQR)	0 [0, 0]	0 [0, 0]	0 (0 to 0)
Fluid volume categories – no. (%)[a]			
Total crystalloid (bolus and maintenance)			
<60 mL/kg	1,214 (29)	1,172 (28)	0.011 (-0.009 to 0.03)
60 to 100 mL/kg	1,397 (33)	1,354 (32)	0.011 (-0.009 to 0.031)
>100 mL/kg	1,621 (38)	1,715 (40)	-0.021 (-0.042 to -0.001)
Bolus fluid only – no. (%)			
<60 mL/kg	3,496 (83)	3,444 (81)	0.014 (-0.0024 to 0.03)

60 to 100 mL/kg	676 (16)	736 (17)	-0.014 (-0.030 to 0.002)
>100 mL/kg	60 (1.4)	61 (1.4)	-0.001 (-0.005 to 0.005)
Protocol Adherence			
No. (%) who received any crystalloid after randomization	4,128 (97)	4,147 (98)	-0.002 (-0.008 to 0.005)
Received ≥75% of total crystalloid as randomized fluid type during intervention phase – no./total no. receiving any crystalloid after randomization (%)	3,300/4,128 (80)	3,661/4,147 (88)	-0.083 (-0.099 to -0.068)

[a] The absolute difference reports the estimated difference in medians calculated as balanced fluids minus 0.9% saline using the Hodges-Lehmann asymptotic estimator or the difference in proportions for categorical variables

[b] Includes fluid administered prior to randomization and during intervention phase

[c] Fluid volume data were missing from 6 patients in the 0.9% saline group and 3 patients in the balanced fluid group due to missing measured weight or inability to impute weight due to missing age

Table S11. Sensitivity Analyses for Effectiveness Outcomes

Outcome	Balanced Fluid (No.)	0.9% Saline (No.)	Risk Difference (95% CI)[a]	Effect Measure (95% CI)[b]
**Complete-case analysis**[c]				
Major adverse kidney events within 30 days	4,073	4,068	0.003 (-0.005 to 0.011)	1.1 (0.87 to 1.4)
Death within 30 days	4,073	4,067	0.001 (-0.004 to 0.005)	1.1 (0.68 to 1.6)
New renal replacement therapy	4,073	4,067	-0.001 (-0.005 to 0.002)	0.84 (0.50 to 1.4)
Persistent kidney dysfunction at hospital discharge	4,071	4,068	0.004 (-0.003 to 0.01)	1.2 (0.89 to 1.6)
Death prior to hospital discharge	4,073	4,067	0 (-0.005 to 0.005)	1.0 (0.67 to 1.5)
Death within 90 days[d]	3,739	3,730	0.002 (-0.004 to 0.009)	1.1 (0.83 to 1.5)
Hospital length of stay	4,071	4,066	---	0 (0 to 0)
Hospital-free days out of 28 days	4,071	4,066	---	0 (0 to 0)
**Per-protocol analysis**[e]				
Major adverse kidney events within 30 days	3,139	3,468	0.002 (-0.005 to 0.01)	1.1 (0.80 to 1.5)
Death within 30 days	3,139	3,468	0.0002 (-0.004 to 0.004)	1.0 (0.59,1.7)
New renal replacement therapy	3,139	3,468	-0.002 (-0.005 to 0.002)	0.67 (0.32,1.4)
Persistent kidney dysfunction at hospital discharge	3,138	3,468	0.003 (-0.004 to 0.009)	1.1 (0.79,1.7)
Death prior to hospital discharge	3,139	3,468	-0.0003 (-0.005 to 0.004)	0.95 (0.58,1.6)
Death within 90 days[d]	2,873	3,174	0.0018 (-0.006 to 0.009)	1.1 (0.77,1.5)
Hospital length of stay	3,139	3,467	---	0 (0 to 0)
Hospital-free days out of 28 days	3,139	3,467	---	0 (0 to 0)

[a] The risk difference is reported as the difference in proportions

[b] The effect measure is reported as site-adjusted risk ratios with 95% confidence intervals estimated using the Mantel–Haenszel method, or as median differences with 95% confidence intervals estimated using the Hodges–Lehmann estimator; analyses are not adjusted for variables other than site as covariate balance was maintained between groups

[c] The "complete-case analysis" included patients without missing data for the primary outcome

[d] Mortality within 90 days was missing if data were not available in the study site medical record

[e] The "per-protocol analysis" included patients who 1) received at least 75% of their total crystalloid fluid volume during the intervention phase as the fluid to which they were randomized, 2) were not retrospectively found to have been ineligible for enrollment, and 3) received at least some crystalloid fluid after randomization

Table S12. Sensitivity Analyses for Pre-randomization 0.9% Saline Administration

Effect Modifier	Risk Ratio (95% CI)	*P* Value for Interaction
Pre-randomization 0.9% saline bolus		0.71
Yes	1.08 (0.81 to 1.45)	
No	1.20 (0.79 to 1.82)	
Pre-randomization 0.9% saline per 100 mL	--	0.92

Post-hoc analyses were completed to evaluate whether receipt of a 0.9% saline bolus prior to enrollment (Yes/No) or the volume of 0.9% saline bolus received prior to enrollment (per 100 mL) modified the treatment effect of MAKE30 for patients randomized to balanced fluid compared to 0.9% saline. Site-adjusted risk ratios were estimated using Poisson regression with a log link and robust standard errors within each subgroup. Effect modification was assessed using treatment-by-subgroup interaction terms. There was no evidence that receipt of 0.9% saline prior to enrollment modified the treatment effect (risk ratio among patients receiving pre-randomization 0.9% saline, 1.08; 95% CI, 0.81 to1.45; risk ratio among patients not receiving pre-randomization 0.9% saline, 1.20, 95% CI, 0.79 to 1.82; P value for interaction=0.71). Similarly, the volume of pre-randomization 0.9% saline bolus fluid administered prior to enrollment did not modify the treatment effect (P value for interaction = 0.92).

Table S13: Median and Range of Laboratory Values by Treatment Arm

Laboratory Measure[a,b]	Balanced Fluid (n=4,235)		0.9% Saline (n=4,247)		*P*[d]
	N	min/25th/50th/75th/max	N	min/25th/50th/75th/max	
Initial sodium, mEq/L	3,892	110/134/**136**/139/199	3,897	104/134/**136**/139/194	0.64
Lowest sodium, mEq/L	2,784	87/135/**137**/139/180	2,828	114/135/**137**/140/162	<0.001
Highest sodium, mEq/L	2,830	125/138/**140**/143/199	2,882	128/138/**140**/143/198	<0.001
Initial potassium, mEq/L	3,822	1.8/3.7/**4.1**/4.5/11	3,808	1.4/3.7/**4.1**/4.5/11	0.87
Highest potassium, mEq/L	2,798	1.3/3.7/**4.1**/4.5/9.1	2,853	2.0/3.7/**4.1**/4.5/12	0.52
Initial chloride, mEq/L	3,741	51/100/**103**/106/163	3,724	59/100/**103**/106/153	0.56
Highest chloride, mEq/L	2,765	91/105/**108**/111/166	2,823	17/107/**110**/114/156	<0.001
Initial bicarbonate, mEq/L	3,762	1.0/19/**21**/24/50	3,770	4.0/19/**21**/24/45	0.23
Lowest bicarbonate, mEq/L	2,644	1.0/19/**22**/24/45	2,723	3.0/18/**20**/23/37	<0.001
Initial creatinine, mg/dL	3,744	0/0.30/**0.40**/0.60/7.6	3,758	0/0.30/**0.40**/0.60/8.8	0.14
Highest creatinine, mg/dL	2,714	0/0.27/**0.40**/0.60/7.2	2,779	0.024/0.30/**0.40**/0.60/9.4	0.28
Last measured creatinine, mg/dL[c]	3,784	0.011/0.23/**0.32**/0.47/6.0	3,812	0.020/0.23/**0.32**/0.46/5.4	0.76
Initial total calcium, mg/dL	3,191	3.6/8.8/**9.2**/9.7/13	3,214	3.7/8.7/**9.2**/9.7/13	0.14
Highest total calcium, mg/dL	2,396	1.0/8.4/**8.9**/9.3/13	2,470	2.0/8.4/**8.8**/9.2/15	<0.001
Initial ionized calcium, mmol/L	2,250	0.70/1.2/**1.2**/1.3/1.8	2,237	0.47/1.1/**1.2**/1.3/1.6	0.50
Highest ionized calcium, mmol/L	1,189	0.63/1.2/**1.2**/1.3/2.3	1,249	0.80/1.2/**1.2**/1.3/2.6	0.19
Initial magnesium, mg/dL	1,283	0.60/1.8/**2.0**/2.2/4.9	1,290	0.90/1.8/**2.0**/2.2/4.0	0.83
Highest magnesium, mg/dL	1,618	1.0/1.8/**2.0**/2.2/5.3	1,713	1.0/1.8/**2.0**/2.2/5.5	0.10
Initial lactate, mmol/L	2,601	0.067/1.3/**1.9**/3.0/23	2,595	0/1.3/**1.9**/3.0/20	0.56
Highest lactate, mmol/L	1,314	0.11/1.3/**2.0**/3.5/58	1,363	0.12/1.2/**1.8**/3.2/35	0.01

Laboratory Measure[a,b]	Balanced Fluid (n=4,235)		0.9% Saline (n=4,247)		P^d
	N	min/25th/50th/75th/max	N	min/25th/50th/75th/max	
Initial alanine aminotransferase, IU/L	2,943	1.0/15/**23**/42/3,300	2,945	0/16/**24**/47/7,180	0.01
Highest alanine aminotransferase, IU/L	1,416	0/16/**28**/68/8,308	1,510	1.0/17/**31**/68/14,000	0.03

[a] Initial laboratory values were values measured closet to randomization (between 6 hours before through 2 hours after randomization)

[b] Highest and lowest laboratory values were the values available from clinician-ordered measurements from the time period between 2 hours after randomization through study day 3.

[c] Last measured creatinine was the last value available from clinician-ordered measurements prior to hospital discharge or study day 30, whichever came first

[d] P values are from the Van Elteren test for continuous outcomes stratified by study site, without adjustment for multiple comparisons

Table S14. Sensitivity Analyses for Safety Outcomes

Safety Outcome	Primary Analysis		Best-case Assumption (Missing = No Event)[a]		Worst-case Assumption (Missing = Event)[b]	
	Risk Ratio (95% CI)	P value	Risk Ratio (95% CI)[c]	P value	Risk Ratio (95% CI)[c]	P value
Sodium >155 mEq/L	0.60 (0.43 to 0.84)	0.003	0.59 (0.43 to 0.83)	0.002	1.00 (0.95 to 1.06)	0.87
Sodium <128 mEq/L	---	---	10.94 (0.11 to 1,062)	0.31	1.03 (0.97 to 1.10)	0.27
Chloride >110 mEq/L	0.64 (0.60 to 0.69)	<0.001	0.63 (0.59 to 0.68)	<0.001	0.84 (0.81 to 0.86)	<0.001
Potassium >6 mEq/L	0.88 (0.63 to 1.20)	0.45	0.86 (0.62 to 1.19)	0.36	1.02 (0.97 to 1.08)	0.40
Calcium total >12 mg/dL or ionized >1.35 mmol/L	0.94 (0.76 to 1.20)	0.60	0.92 (0.73 to 1.16)	0.47	1.02 (1.00 to 1.04)	0.09
Lactate >4 mmol/L	1.20 (1.0 to 1.40)	0.04	1.15 (0.97 to 1.36)	0.11	1.02 (1.00 to 1.05)	0.05

[a] All participants with missing laboratory values were assumed not to have met the safety threshold.

[b] All participants with missing laboratory values were assumed to have met the safety threshold.

[c] Site-adjusted risk ratios with 95% confidence intervals and P values are estimated using the Cochrane-Mantel–Haenszel method

Table S15. Adverse Events

Adverse Event	Balanced Fluid	0.9% Saline	P Value
Any adverse event through study day 7– n (%)	1,652 (39)	1,876 (44)	<0.001
Death – n (%)	25 (0.59)	26 (0.61)	0.90
Cardiac arrest requiring CPR – n (%)	21 (0.50)	23 (0.54)	0.77
Arrhythmia requiring intervention – n (%)	11 (0.26)	14 (0.33)	0.55
Extracorporeal membrane oxygenation – n (%)	28 (0.66)	18 (0.42)	0.14
Invasive mechanical ventilation – n (%)	355 (8.4)	356 (8.4)	0.99
Non-invasive mechanical ventilation – n (%)	385 (9.1)	391 (9.2)	0.86
Acute kidney injury, fluid overload, acidemia, or electrolyte abnormality requiring dialysis – n (%)	25 (0.59)	30 (0.71)	0.51
Liver dysfunction – n (%)	186 (4.4)	176 (4.2)	0.57
Limb necrosis – n (%)	9 (0.21)	8 (0.19)	0.80
Brain herniation, in absence of CNS, mastoid, or sinus infection – n (%)	2 (0.05)	3 (0.07)	0.66
Brain herniation, in presence of CNS, mastoid, or sinus infection – n (%)	3 (0.07)	7 (0.17)	0.21
Seizure, in absence of CNS infection – n (%)	42 (0.99)	46 (1.1)	0.68
Seizure, in presence of known/suspected CNS infection or in subject with history of seizures – n (%)	96 (2.3)	102 (2.4)	0.68
Pulmonary embolus – n (%)	6 (0.14)	8 (0.19)	0.60
Deep venous thrombosis – n (%)	36 (0.85)	35 (0.83)	0.89

Central venous line thrombosis requiring pharmacologic therapy – n (%)	20 (0.47)	15 (0.35)	0.39
Bleeding (not requiring massive transfusion protocol) – n (%)	20 (0.47)	24 (0.57)	0.55
Bleeding (requiring massive transfusion protocol) – n (%)	5 (0.12)	4 (0.09)	0.74
Hospital-acquired infection – n (%)	27 (0.64)	38 (0.90)	0.17
Intravenous infiltrate (grade 3 or 4) – n (%)	24 (0.57)	38 (0.90)	0.08
Hyperlacatatemia >4 mmol/L – n (%)	272 (6.4)	237 (5.6)	0.10
Hyperkalemia >6 mEq/L – n (%)	57 (1.3)	76 (1.8)	0.10
Hypercalcemia (total calcium >12 mEq/L or ionized calcium >1.35 mEq/L or) – n (%)	89 (2.1)	82 (1.9)	0.57
Hypernatremia >155 mEq/L – n (%)	52 (1.2)	88 (2.1)	0.002
Hyponatremia <125 mEq/L – n (%)	23 (0.54)	19 (0.45)	0.53
Hyperchloremia >110 mEq/L – n (%)	835 (20)	1,241 (29)	<0.001
Other – n (%)	76 (1.8)	76 (1.8)	0.98
Any serious adverse event through study day 7 – n (%)	25 (1.5)	26 (1.4)	0.80

CNS, central nervous system

Table S16. Representativeness of Study Participants

Category	Example
Disease, problem, or condition under investigation	Children (ages 2 months to <18 years) treated for community-acquired septic shock, defined as sepsis with abnormal perfusion
Special considerations related to	
Sex	Sepsis/septic shock affects slightly more male than female children (ratio ~ 1.1: 1) but mortality is not different by sex.[20-23]
Age	Sepsis/septic shock has a bimodal distribution in children, with the highest rates among infants/preschool children 0-5 years-old (especially neonates < 1 month-old) and adolescents 12-18 years-old.[20-22,24]
Race or ethnic group	The racial/ethnic distribution among children with sepsis/septic shock typically reflects the source population.[20-23,25] Although minority groups in high-resource countries have been reported to have higher mortality from sepsis/septic shock than children identified as White or non-Hispanic in some reports, this risk is more closely linked to socioeconomic factors.[25]
Geography	Children living in low-resource countries have a higher incidence of and mortality from sepsis/septic shock than children in high-resource countries.[26]
Other considerations	Septic shock refers to the subset of patients with sepsis who have cardiovascular dysfunction. For children, cardiovascular was defined as "abnormal perfusion" by the 2005 International Pediatric Sepsis

	Consensus Conference[27] until the more recent 2024 Phoenix criteria established new criteria to identify a subset of patients with higher mortality.[28] However, the term septic shock has long been and continues to remain broadly applicable in clinical practice as an infection with concurrent abnormalities in perfusion for the purposes of early recognition and treatment in children.[29-31] Community-acquired sepsis/septic shock is 2 to 4 times more common than hospital-acquired sepsis/septic shock among children, but mortality is lower (1-3% versus 8-20%).[32] The most frequent sites of infection leading to sepsis/septic shock in children are respiratory, abdominal, bacteremia, and genitourinary.[20,21] Blood cultures positive for a microbial pathogen are reported in 10-30% of children with community-acquired sepsis/septic shock, while higher rates are reported in hospital-acquired sepsis/septic shock and those children in the intensive care unit).[20,21,33-35] Children with chronic comorbid conditions, especially neurologic disorders and cancer, are especially vulnerable to developing sepsis/septic shock with an overall prevalence of chronic comorbid conditions among children with sepsis/septic shock between 10% and 80% across studies.[20,32,35]
Overall representativeness of this trial	The children in this trial were representative of community-acquired septic shock in high-resource settings. The term "septic shock" was applied as it is commonly used in clinical practice to identify children with infection and abnormal perfusion, though not all enrolled patients attained the 2024 Phoenix criteria for septic shock. Patients in our study demonstrated a slightly older age distribution than epidemiologic reports because the highest-risk neonatal age group was excluded from enrollment, although the expected adolescent age peak was preserved. Biologic sex exhibited the expected slight male predominance (51%).

	The proportion of patients with Black, Asian, and American Indian race and Hispanic ethnicity mirrored the United States population, as did the proportion with Aboriginal/Torres Strait Islander ethnicity in Australia and Māori ethnicity in New Zealand (race was not authorized to be collected at Canadian sites). Where obtained, race was self-reported. Sites of infection (most commonly, respiratory) were consistent with epidemiologic data for children with community-acquired sepsis/septic shock, as was the rate of primary/secondary bacteremia (12%). The presence of key comorbid conditions, including neurologic disorders (28%) and cancer (14%), were as expected based on prior studies. Children living in low-resource countries and those with hospital-acquired sepsis/septic shock were not represented in this study.

SUPPLEMENTAL REFERENCES

1. Zwarenstein M, Treweek S, Gagnier JJ, et al. Improving the reporting of pragmatic trials: an extension of the CONSORT statement. BMJ 2008;337:a2390.
2. Baren JM, Fish SS. Resuscitation research involving vulnerable populations: are additional protections needed for emergency exception from informed consent? Acad Emerg Med 2005;12(11):1071-7.
3. Chamberlain JM, Okada P, Holsti M, et al. Lorazepam vs diazepam for pediatric status epilepticus: a randomized clinical trial. JAMA 2014;311(16):1652-60.
4. Inwald DP, Canter R, Woolfall K, et al. Restricted fluid bolus volume in early septic shock: results of the Fluids in Shock pilot trial. Arch Dis Child 2018.
5. Dalziel SR, Borland ML, Furyk J, et al. Levetiracetam versus phenytoin for second-line treatment of convulsive status epilepticus in children (ConSEPT): an open-label, multicentre, randomised controlled trial. Lancet 2019;393(10186):2135-2145.
6. Balamuth F, Kittick M, McBride P, et al. Pragmatic pediatric trial of balanced versus normal saline fluid in sepsis: the PRoMPT BOLUS pilot feasibility study. Acad Emerg Med 2019;26(12):1346-56.
7. Watters D, Sayre MR, Silbergleit R. Research conditions that qualify for emergency exception from informed consent. Acad Emerg Med 2005;12(11):1040-4.
8. Haggins AN, Harney D, Scott S, Silbergleit R. A systematic review of Federal Drug Administration Docket for community consultation and public disclosure in exception from informed consent trials. Clin Trials 2018;15(1):29-35.
9. Harvin JA, Podbielski JM, Vincent LE, et al. Impact of Social Media on Community Consultation in Exception From Informed Consent Clinical Trials. J Surg Res 2019;234:65-71.

10. National Statement on Ethical Conduct in Human Research. The National Health and Medical Research Council tARCaUA. Canberra: Commonwealth of Australia, 2018. (https://www.nhmrc.gov.au/about-us/publications/national-statement-ethical-conduct-human-research-2007-updated-2018).
11. Tri-Council Policy Statement: Ethical Conduct for Research Involving Humans — TCPS 2. Canadian Institutes of Health Research NSaERCoC, & Social Sciences and Humanities Research Council of Canada. Canada: Government of Canada, 2022. (https://www.pre.ethics.gc.ca/eng/documents/tcps2-2022-en.pdf).
12. Raman S, Gibbons KS, Mattke A, et al. Effect of Saline vs Gluconate/Acetate-Buffered Solution vs Lactate-Buffered Solution on Serum Chloride Among Children in the Pediatric Intensive Care Unit: The SPLYT-P Randomized Clinical Trial. JAMA Pediatr 2022;177(2):122-31.
13. Weiss SL, Peters MJ, Alhazzani W, et al. Surviving Sepsis Campaign International Guidelines for the Management of Septic Shock and Sepsis-Associated Organ Dysfunction in Children. Pediatr Crit Care Med 2020;21(2):e52-e106.
14. Young JB, Utter GH, Schermer CR, et al. Saline versus Plasma-Lyte A in initial resuscitation of trauma patients: a randomized trial. Ann Surg 2014;259(2):255-62.
15. Kuczmarski RJ, Ogden CL, Grummer-Strawn LM, et al. CDC growth charts: United States. Adv Data 2000(314):1-27.
16. Weiss SL, Balamuth F, Thurm CW, Downes KJ, Fitzgerald JC, Laskin BL. Major Adverse Kidney Events in Pediatric Sepsis. Clin J Am Soc Nephrol 2019;14(5):664-72.
17. Self WH, Semler MW, Wanderer JP, et al. Balanced Crystalloids versus Saline in Noncritically Ill Adults. N Engl J Med 2018;378(9):819-28.
18. Semler MW, Self WH, Wanderer JP, et al. Balanced Crystalloids versus Saline in Critically Ill Adults. N Engl J Med 2018;378(9):829-39.

19. Palevsky PM, Molitoris BA, Okusa MD, et al. Design of clinical trials in acute kidney injury: report from an NIDDK workshop on trial methodology. Clin J Am Soc Nephrol 2012;7(5):844-50.

20. Weiss SL, Fitzgerald JC, Pappachan J, et al. Global Epidemiology of Pediatric Severe Sepsis: the Sepsis PRevalence, OUtcomes, and Therapies Study. Am J Respir Crit Care Med 2015;191(10):1147-57.

21. Hartman ME, Linde-Zwirble WT, Angus DC, Watson RS. Trends in the Epidemiology of Pediatric Severe Sepsis. Pediatr Crit Care Med 2013;14(7):868-93.

22. Balamuth F, Scott HF, Weiss SL, et al. Validation of the Pediatric Sequential Organ Failure Assessment Score and Evaluation of Third International Consensus Definitions for Sepsis and Septic Shock Definitions in the Pediatric Emergency Department. JAMA Pediatr 2022;176(7):672-8.

23. Schlapbach LJ, Straney L, Alexander J, et al. Mortality related to invasive infections, sepsis, and septic shock in critically ill children in Australia and New Zealand, 2002-13: a multicentre retrospective cohort study. Lancet Infect Dis 2015;15(1):46-54.

24. Singhal S, Allen MW, McAnnally JR, Smith KS, Donnelly JP, Wang HE. National estimates of emergency department visits for pediatric severe sepsis in the United States. PeerJ 2013;1:e79.

25. Li E, Ng AP, Williamson CG, Tran Z, Federman MD, Benharash P. Assessment of Racial and Ethnic Disparities in Outcomes of Pediatric Hospitalizations for Sepsis Across the United States. JAMA Pediatr 2023;177(2):206-8.

26. Rudd KE, Johnson SC, Agesa KM, et al. Global, regional, and national sepsis incidence and mortality, 1990-2017: analysis for the Global Burden of Disease Study. Lancet 2020;395(10219):200-11.

27. Goldstein B, Giroir B, Randolph A. International pediatric sepsis consensus conference: definitions for sepsis and organ dysfunction in pediatrics. Pediatr Crit Care Med 2005;6(1):2-8.

28. Schlapbach LJ, Watson RS, Sorce LR, et al. International Consensus Criteria for Pediatric Sepsis and Septic Shock. JAMA 2024;331(8):665-74.

29. Fisher JD, Nelson DG, Beyersdorf H, Satkowiak LJ. Clinical spectrum of shock in the pediatric emergency department. Pediatr Emerg Care 2010;26(9):622-5.

30. Georgette N, Sheehan M, Kissoon N. Perspectives on the Phoenix Sepsis Criteria for the Emergency Medicine Practitioner. Pediatr Emerg Care 2026;42(2):146-53.

31. Morin L, Hall M, de Souza D, et al. The Current and Future State of Pediatric Sepsis Definitions: An International Survey. Pediatrics 2022;149(6):e2021052565.

32. Weiss SL, Balamuth F, Chilutti M, et al. Identification of Pediatric Sepsis for Epidemiologic Surveillance Using Electronic Clinical Data. Pediatr Crit Care Med 2020;21(2):113-21.

33. Scott HF, Brilli RJ, Paul R, et al. Evaluating Pediatric Sepsis Definitions Designed for Electronic Health Record Extraction and Multicenter Quality Improvement. Crit Care Med 2020;48(10):e916-26.

34. Matics TJ, Sanchez-Pinto LN. Adaptation and Validation of a Pediatric Sequential Organ Failure Assessment Score and Evaluation of the Sepsis-3 Definitions in Critically Ill Children. JAMA Pediatr 2017;171(10):e172352.

35. Ruth A, McCracken CE, Fortenberry JD, Hall M, Simon HK, Hebbar KB. Pediatric Severe Sepsis: Current Trends and Outcomes From the Pediatric Health Information Systems Database. Pediatr Crit Care Med 2014;15(9):828-38.
